# Computational Analysis of Targeting SARS-CoV-2, Viral Entry Proteins ACE2 and TMPRSS2, and Interferon Genes by Host MicroRNAs

**DOI:** 10.3390/genes11111354

**Published:** 2020-11-16

**Authors:** Jacob B. Pierce, Viorel Simion, Basak Icli, Daniel Pérez-Cremades, Henry S. Cheng, Mark W. Feinberg

**Affiliations:** 1 Department of Medicine, Cardiovascular Division, Brigham and Women’s Hospital, Harvard Medical School, Boston, MA 02115, USA; Jacob.pierce@northwestern.edu (J.B.P.); simiond.viorel@gmail.com (V.S.); BICLI@BWH.HARVARD.EDU (B.I.); dperez-cremades@bwh.harvard.edu (D.P.-C.); sccheng@bwh.harvard.edu (H.S.C.); 2 Feinberg School of Medicine, Northwestern University, Chicago, IL 60611, USA

**Keywords:** SARS-CoV-2, COVID-19, microRNA, ACE2, TMPRSS2

## Abstract

Rapid spread of severe acute respiratory syndrome coronavirus 2 (SARS-CoV-2), the virus responsible for coronavirus disease 2019 (COVID-19), has led to a global pandemic, failures of local health care systems, and global economic recession. MicroRNAs (miRNAs) have recently emerged as important regulators of viral pathogenesis, particularly among RNA viruses, but the impact of host miRNAs on SARS-CoV-2 infectivity remains unknown. In this study, we utilize the combination of powerful bioinformatic prediction algorithms and miRNA profiling to predict endogenous host miRNAs that may play important roles in regulating SARS-CoV-2 infectivity. We provide a collection of high-probability miRNA binding sites within the SARS-CoV-2 genome as well as within mRNA transcripts of critical viral entry proteins ACE2 and TMPRSS2 and their upstream modulators, the interferons (IFN). By utilizing miRNA profiling datasets of SARS-CoV-2-resistant and -susceptible cell lines, we verify the biological plausibility of the predicted miRNA–target RNA interactions. Finally, we utilize miRNA profiling of SARS-CoV-2-infected cells to identify predicted miRNAs that are differentially regulated in infected cells. In particular, we identify predicted miRNA binders to SARS-CoV-2 ORFs (miR-23a (1ab), miR-29a, -29c (1ab, N), miR-151a, -151b (S), miR-4707-3p (S), miR-298 (5′-UTR), miR-7851-3p (5′-UTR), miR-8075 (5′-UTR)), ACE2 3′-UTR (miR-9-5p, miR-218-5p), TMPRSS2 3′-UTR (let-7d-5p, -7e-5p, miR-494-3p, miR-382-3p, miR-181c-5p), and IFN-α 3′-UTR (miR-361-5p, miR-410-3p). Overall, this study provides insight into potential novel regulatory mechanisms of SARS-CoV-2 by host miRNAs and lays the foundation for future investigation of these miRNAs as potential therapeutic targets or biomarkers.

## 1. Introduction

Since December 2019, coronavirus disease 2019 (COVID-19) has rapidly spread from Wuhan, China across the globe, with nearly four million confirmed cases and two-hundred and fifty thousand deaths as of May, 2020, receiving designation as a global pandemic by the World Health Organization [1,2]. A novel betacoronavirus termed severe acute respiratory syndrome coronavirus 2 (SARS-CoV-2) has been identified as the infectious agent responsible for COVID-19 [3]. Infection with SARS-CoV-2 may lead to a wide variety of clinical syndromes primarily involving the respiratory tract, ranging from asymptomatic infection to acute respiratory distress syndrome and death [4,5]. While many clinical trials are ongoing, there are currently minimal established therapies to prevent or treat COVID-19, highlighting the urgent need for further mechanistic insights into the cellular mechanisms underlying SARS-CoV-2 infection.

Much like other betacoronaviruses such as SARS-CoV and MERS-CoV, SARS-CoV-2 is a single-strand, positive-sense RNA virus that uses its viral spike glycoprotein (S protein) to bind to specific target-cell surface receptors and initiate viral entry [6]. Similar to SARS-CoV, SARS-CoV-2 binds specifically to host-cell angiotensin-converting enzyme 2 (ACE2), after which host serine protease TMPRSS2 cleaves the S protein to facilitate membrane fusion [7,8,9,10]. Several recent analyses of single-cell RNA sequencing datasets have demonstrated that co-expression of both ACE2 and TMPRSS2 occurs in only a minority of cells, primarily secretory epithelial cells in the upper respiratory tract [11,12,13], suggesting that cell type-specific mechanisms may be important for SARS-CoV-2 infection and propagation. Similarly, commonly used human cell lines have demonstrated varying degrees of infectivity with SARS-CoV-2 [14], although the mechanisms governing cell line-specific phenotypes remain unknown. Identifying host factors that influence ACE2 and TMPRSS2 expression may provide crucial insights into cellular mechanisms that might be therapeutically exploited against SARS-CoV-2 infection.

MicroRNAs (miRNAs) are well-conserved, short (20–22 nt) non-coding RNA molecules that regulate a broad array of cellular functions at the post-transcriptional level. Cellular miRNAs play an important role in the regulation of gene expression in eukaryotes. Canonical gene regulation by miRNAs involves the binding of miRNAs to the 3′ untranslated region (UTR) of target messenger RNA (mRNA) transcripts, promoting translational repression and mRNA degradation [15]. As a post-transcriptional gene expression regulatory molecule, miRNAs act as key effector molecules in the complicated interaction network between virus and host. Both virus and host can encode miRNAs [16]. Virus miRNAs can resist human cell antiviral immune defense systems by changing various host gene expression to control cell growth and development [17,18]. In contrast, cell-encoded miRNAs can directly affect viral entry and replication cycle [19]. In addition, miRNAs can affect viral infectivity indirectly by regulating host factors involved with viral pathogenesis [19]. Therefore, studies on host cell-derived miRNAs can contribute to the further understanding of mechanisms underlying the interactions between virus and host cells and provide a framework for the discovery of novel antiviral agents and strategies. For example, miRNAs have been shown to target and regulate viral gene expression by targeting different regions of the viral genome, including structural (membrane, envelope, nucleocapsid), non-structural proteins, or the non-coding (untranslated) regions [19]. However, the role of miRNAs in dictating SARS-CoV-2 pathogenesis is unknown.

To address these knowledge gaps, we investigated potential interactions between host miRNAs and SARS-CoV-2. The goals of our study were 2-fold: (1) identify host miRNAs that may directly bind to SARS-CoV-2 genomic RNA and thus affect viral infectivity; and (2) identify host miRNAs that may regulate ACE2 and TMPRSS2 expression, or their upstream interferon (IFN) regulators, and thus control the susceptibility of cells to infection by SARS-CoV-2. By utilizing bioinformatic prediction algorithms to predict miRNA binding to viral and host RNA transcripts in combination with RNA-sequencing data in cell lines with known resistance or susceptibility to SARS-CoV-2 infection, we provide an atlas of miRNAs that are predicted to bind to the SARS-CoV-2 genome directly and thus potentially affect genomic stability, translation, or replication. We also describe host miRNAs that may regulate ACE2, TMPRSS2, or IFN-α, -β, or -γ expression. Finally, we use miRNA profiling from SARS-CoV-2-infected cells to investigate changes in expression of predicted miRNAs following infection. These data establish a detailed framework for future mechanistic studies investigating the relationship of these important miRNA host cell–SARS-CoV-2 interactions and provide a foundation for potential new targets for therapy.

## 2. Materials and Methods

### 2.1. SARS-CoV-2 Genomic Analysis

National Center for Biotechnology Information (NCBI) Virus was queried for SARS-CoV-2 complete genomes deposited with NCBI through 19 April 2020. Five genomes were excluded due to poor sequence quality, resulting in 820 unique SARS-CoV-2 genomes used in further analyses. Viral genomic sequences were aligned using MUltiple Sequence Comparison by Log-Expectation (MUSCLE) [20], and a rooted phylogenetic tree was constructed by the unweighted pair group method with six base nucleotide k-mers [21] and visualized using iTOL [22]. NCBI Basic Local Alignment Search Tool (BLAST) [23] was used to investigate genomic sequence identity. SARS-CoV-2 isolate Wuhan-Hu-1 (GenBank accession: MN908947.3) was used as a reference sequence similar to previous phylogenetic analyses [24,25].

### 2.2. MiRNA Binding Site Prediction

MiRNA binding sites within the SARS-CoV-2 genome were predicted using miRDB (http://mirdb.org/index.html) [26]. Similar to previous analyses [27], candidate miRNAs with a miRDB target score ≥70 were retained for further analysis. RNAhybrid (https://bio.tools/rnahybrid) [28] was used to verify miRDB-predicted miRNA–SARS-CoV-2 genomic binding. RNAhybrid queries specified seed region binding (specified as nucleotides 2–8 on candidate miRNAs), a minimum energy threshold of -18 Kcal/mol, and a maximum bulge and internal loop length of 1. The insertion of a polybasic amino acid sequence (12 nt) in the SARS-CoV-2 S protein has been implicated in the unique pathogenic properties of SARS-CoV-2 [25]. To maximize the sensitivity for potential miRNA binding sites, the stringency of the miRDB target score and RNAhybrid minimum energy thresholds were relaxed for a dedicated analysis of this genomic region. Candidate miRNAs meeting the above inclusion criteria of both miRDB and RNAhybrid databases were retained for expression analyses. RNA secondary structure predictions were performed using RNAfold http://rna.tbi.univie.ac.at/cgi-bin/RNAWebSuite/RNAfold.cgi) and presented as the predicted minimum free energy conformation [29]. MiRWalk 2.0 (http://mirwalk.umm.uni-heidelberg.de/) [30] was used to predict miRNA binding sites in the 3′ UTR of viral entry proteins ACE2 and TMPRSS2 as well as IFN-α, -β, and -γ as ACE2 has recently been shown to be an IFN-responsive gene [11].

### 2.3. MiRNA Expression in Human Tissue and Cell Lines

The expression of candidate miRNAs predicted to bind both the viral genome as well as mRNA transcripts of proteins of interest (ACE2, TMPRSS2, and IFNs) was analyzed in human tissue samples to verify the biologic plausibility of binding interactions. The Human MicroRNA Tissue Atlas (https://ccb-web.cs.uni-saarland.de/tissueatlas/) was used to investigate candidate miRNA expression in human tissue samples [31]. Tissues analyzed in the Human MicroRNA Tissue Atlas project originated from two male subjects. The first was from a 65-year-old male patient who died due to multiple myeloma. The body was stored at 4 °C after arrival at the anatomical institute and dissected 2 days post-mortem. The second was from a 59-year-old male individual, who died a natural death. The body was frozen at −20 °C after arrival at the anatomical institute and dissected after 3 weeks of storage. Autopsy showed no signs of cancer. Lung, liver, kidney, and lymph nodes were amongst the 37 types of tissues collected from both. MiRNA expression was also investigated in several commonly used cell lines. Cell lines were chosen based on proven infectivity with SARS-CoV-2 as determined by post-inoculation viral titers, copy number quantification, luciferase reporter assays, and their reported expression of ACE2 and TMPRSS2. Huh7 [8,14,32,33] and Calu3 [8,14,32,34] were evaluated as cell lines with high SARS-CoV-2 infectivity, and A549 [8,14,32,33] and primary human lung fibroblasts (LF) [12,13] were evaluated as cells with low infectivity. Changes in miRNA expression following SARS-CoV-2 infection were based on miRNA expression at 24 h post-inoculation with either SARS-CoV-2 or mock [35].

### 2.4. Statistical Analysis

Absolute miRNA expression in human lung tissue and cell lines was expressed in copies per million (CPM). MiRNA expression profiles of Huh7, Calu-3, A549 and human primary lung fibroblasts were downloaded from https://www.ncbi.nlm.nih.gov/geoprofiles/. MiRNA profiling of untreated Huh7 and A549 was performed as a part of the same experiment (GSE116179). We therefore directly compared miRNA profiles between these two cell lines with high and low SARS-CoV-2 infectivity, respectively [36]. Relative expression between Huh7 and A549 was expressed as log2-fold change in Huh7 relative to A549. Differential expression analyses were performed using DESeq2 R package [37]. MiRNA profiling of Calu-3 (GSE139516) [38] and LF (GSE125183) [39] was performed as a part of independent experiments, limiting direct statistical comparisons between these cell lines. However, miRNAs that play important roles in the susceptibility or resistance to SARS-CoV-2 infection will likely display similar relative expression across cell lines (i.e., miRNAs important to viral susceptibility will likely be more highly expressed relative to other miRNAs in both Huh7 and Calu-3 and lowly expressed in A549 and LF) [40]. We therefore conducted semi-quantitative analyses in which we divided miRNAs into terciles of relative expression within each cell line, allowing comparison across all four cell lines. Relative expression of RNAseq data from Calu-3 cells infected with SARS-CoV-2 was conducted using DESeq2 R package and calculated as log2-fold change in Calu-3 cells infected for 24 h with SARS-CoV-2 compared to mock-infected cells (GSE148729). Adjusted *p* values calculated from the DESeq2 R package were used in our analyses. Student *t*-test was used to determine statistical significance between groups. Two-tailed *p* < 0.05 was considered statistically significant. Statistical analysis and data visualization were performed in STATA/IC software version 15.1 (College Station, TX, USA), GraphPad Prism version 7.0a (La Jolla, CA), and R version 3.6.2 (Vienna, Austria).

## 3. Results

### 3.1. Globally Representative SARS-CoV-2 Genomes Demonstrate Minimal Variation in Nucleotide Sequence and Predicted miRNA Binding Sites

**Figure 1:**
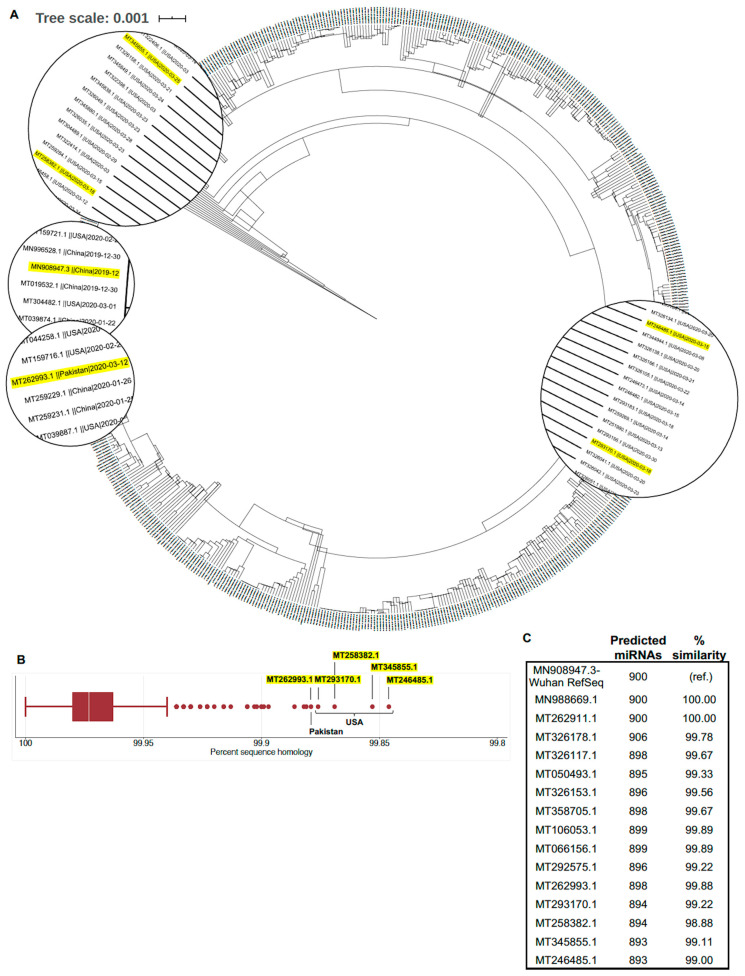
Phylogenetic analysis of severe acute respiratory syndrome coronavirus 2 (SARS-CoV-2) genomes demonstrate minimal sequence variation. (**A**) Phylogenetic tree of 820 SARS-CoV-2 complete genomes deposited in the National Center for Biotechnology Information (NCBI) database through 19 April 2020. Highlighted sequences indicate those selected for further sequence analysis shown in Table 1. (**B**) Percent sequence identity of SARS-CoV-2 complete genomes compared with the reference sequence (MN908947.3). (**C**) Changes in miRDB-predicted microRNA binding sites predicted along the full SARS-CoV-2 genomic sequence for genomes with variable identity.

First, we sought to determine whether a single SARS-CoV-2 genome sequence (i.e., reference sequence MN908947.3) could be used to predict binding by potential hosts miRNAs to SARS-CoV-2 more broadly. Phylogenetic analysis of the 820 included SARS-CoV-2 genomes demonstrated detectable deviation from the SARS-CoV-2 reference sequence (Figure 1A). However, when compared to the SARS-CoV-2 reference sequence using BLAST, there was remarkably little sequence variation in all included sequences (mean percent sequence identity = 99.97%, standard deviation = 0.02%; Figure 1B). To investigate the impact of the small degree of genetic variation between SARS-CoV-2 genomes on potentially important miRNA binding sites, we selected three genomic regions—the 5′ UTR, S protein, and 3′ UTR—of the five sequences with the lowest sequence identity compared to the reference sequence (Table 1). These sites were selected based on previously described interactions between host miRNAs and 5′ and 3′ UTRs of RNA viral genomes [41,42,43] as well as the critical importance of the S protein for SARS-CoV-2 pathogenicity [7,9,10]. Overall, the genetic sequences were highly conserved compared to the reference sequence. Four of these samples originated in the United States, three of which were from Washington state. The fifth sample was from Punjab, Pakistan. The most common cause of sequence deviation from the reference sequence was short “N” repeats (indicating unknown nucleotides due to unreliable sequencing). Point mutations (C–>T) were identified in the 5′-UTRs of SARS-CoV-2 genomes from Washington (MT345855.1) and California (MT258382.1), USA, but overall sequence identity remained at 99.85% and 99.87%, respectively (Table 1). Additional point mutations (T–>W, either T or A; or A–>G) occurred in the S spike protein domain or (G–>R, either G or A; or C–>M, either C or A) in the 3′UTR of the SARS-CoV-2 genome from California (MT258382.1) Truncations to the 5′ UTR, 3′ UTR, or both were common among all five sequences sampled. Additionally, one United States sequence (MT293170.1) contained a 9 nt poly-A insertion within the poly-A tail of the 3′ UTR. Despite the minor detected differences in genomic sequence, there was very little change in predicted miRNA binding sites when the full SARS-CoV-2 genomes were queried in miRDB (Figure 1C). Based on these results, the SARS-CoV-2 reference sequence Wuhan-Hu-1 (GenBank accession: MN908947.3) was selected for use in further miRNA prediction analyses to maintain comparability with previously published studies.

### 3.2. Genomic Regions of SARS-CoV-2 Demonstrate Unique Predicted miRNA Binding Profiles

**Figure 2:**
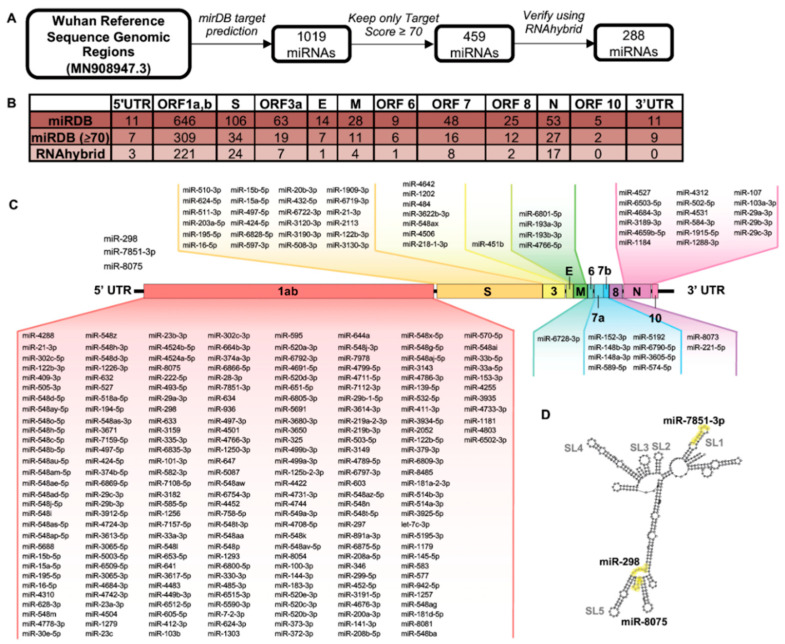
Bioinformatic analysis of the severe acute respiratory syndrome coronavirus 2 (SARS-CoV-2) genome reveals potential microRNA (miRNA) binding sites within distinct genomic regions. (**A**) Open reading frames (ORFs) from the SARS-CoV-2 reference genome (MN908947.3) were probed for potential binding sites of human miRNAs. MiRNAs with a target score ≥70 were validated using RNAhybrid. (**B**) Number of predicted miRNAs binding to individual SARS-CoV-2 genomic regions. Intergenic sequences are included in ORF following intergenic sequence. (**C**) Predicted miRNAs binding to indicated SARS-CoV-2 ORFs. (**D**) Highlighted miRNA binding sites within 5′ UTR. Evolutionarily conserved stem–loop (SL) structures are highlighted.

To predict miRNAs that may bind directly to the SARS-CoV-2 genome, we conducted individual miRDB queries for each open reading frame (ORF) of the SARS-CoV-2 genome as well as the 5′ and 3′ UTRs (1019 total miRNAs; Figure 2A,B). MiRNAs with a miRDB target score ≥70 (459 miRNAs) were then verified using RNAhybrid, resulting in a final collection of 288 miRNAs predicted to bind to the SARS-CoV-2 genome with high probability. Individual miRNAs and their predicted binding sites within the SARS-CoV-2 genome are schematically displayed in Figure 2C. ORF1ab contained the majority of predicted miRNA binding sites (221 of 288 total binding sites) in part due to its longer sequence. The S protein and nucleoprotein (N protein) ORFs contained 24 and 17 miRNA binding sites, respectively. Similarly, the other domains had a reduced number or no miRNA binding sites (5′-UTR (3 sites), ORF3a (7 sites), E (1 site), M (4 sites), ORF 6 (1 site), ORF 7 (8 sites), ORF 8 (2 sites), and ORF 10 (0 sites)). Notably, the 3′ UTR contained no binding sites meeting pre-specified miRDB and RNAhybrid cut offs. The interaction between miRNAs and viral nucleotide secondary structure has previously shown to play important roles in viral pathogenesis, particularly in the viral UTRs [41]. We therefore investigated the binding sites of the three miRNAs predicted to bind the 5′ UTR in the context of its predicted nucleotide secondary structure (Figure 2D). Interestingly, all three miRNAs are predicted to bind either within or adjacent to stem–loop (SL) structures that are highly conserved among other viruses, miR-298, miR-7851-3p, and miR-8075, in the *Coronaviridae* family and known to be important for viral function [44].

### 3.3. Expression of miRNAs in Lungs Predicted to Bind SARS-CoV-2

**Figure 3:**
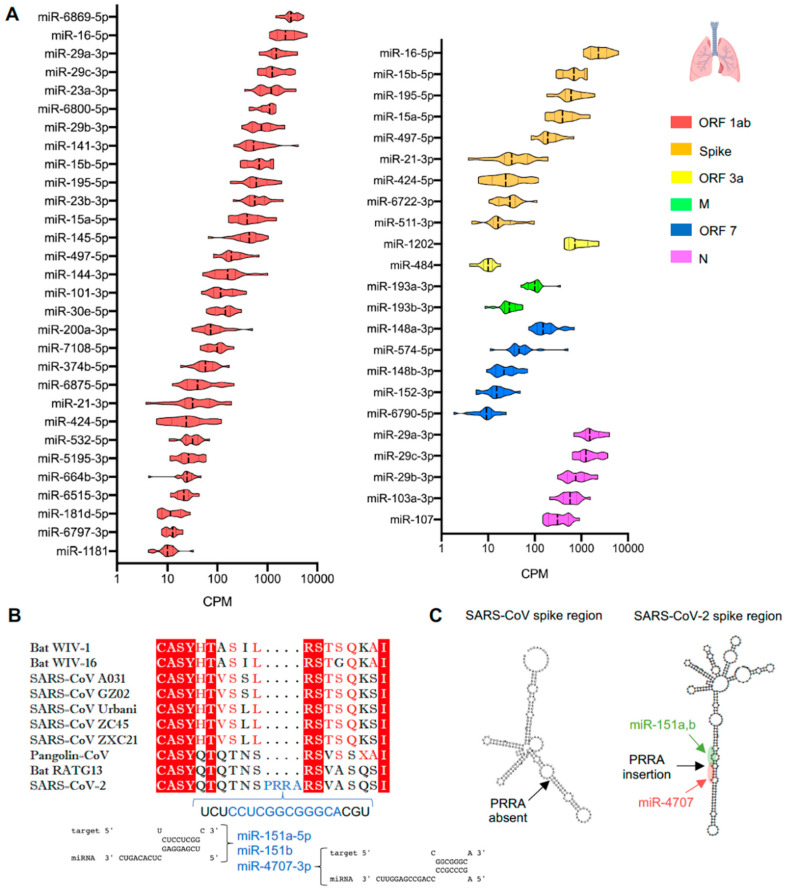
Expression in lungs of microRNAs (miRNAs) targeting the severe acute respiratory syndrome coronavirus 2 (SARS-CoV-2) genome. (**A**) Expression in human healthy lungs of miRNAs predicted to target distinct genomic regions of SARS-CoV-2. (**B**) Amino acid sequence alignment of the spike protein and its phylogenic coronaviruses, and the miRNAs binding in the unique sequence representing the polybasic cleavage site (PRRA). (**C**) Secondary structures of genomic region encoding SARS-CoV and SARS-CoV-2 S proteins in a 200 nt region of the SARS-CoV-2-specific PRRA polybasic cleavage site and highlighted microRNA binding sites within the PRRA region.

We further assessed the lung expression of miRNAs predicted to bind to the SARS-CoV-2 genome using the miRNA Tissue ATLAS database. Of the 288 miRNAs interrogated, 53 miRNAs were expressed in the lungs while the rest were undetectable (Figure 3A). The miR-6869-5p targeting ORF1ab showed the highest expression in the lungs. High expression was also observed for miR-16-5p, -15a-5p, -15b-5p, -195-5p and -497-5p targeting both the ORF1ab and gene encoding the S protein while miR-21-3p and 424-5p showed an average expression in the same regions. The miRNA family miR-29a, -29b, -29c (-3p), which are predicted to bind both ORF1ab and ORF9 encoding the non-structural proteins and nucleocapsid protein, respectively, were highly expressed in the lungs. More specifically, within ORF1ab, miR-29a-3p is predicted to bind to the region encoding Nsp14, and the miR-23a-3p binding site resides in the region encoding Nsp3. MiR-15a and -15b and miR-16 have 7, 8, and 6 binding sites within ORF1ab, respectively. The miRNAs miR-23a and -23b (-3p), predicted to bind the ORF1ab, and miR-1202, predicted to bind the ORF3a region, showed similarly high expression in human lungs. In contrast, relatively low lung expression was observed for miR-193a and -193b (-3p), which target the membrane (M) protein coding region, and for miR-148a and -148b (-3p), which target the ORF7a, b region of SARS-CoV-2. (Figure 3A).

One of the most important domains that defines specificity of the SARS-CoV-2 sequence is the acquisition in the spike (S) domain of a polybasic cleavage site (RRAR) with a leading proline (P), a finding absent in SARS-CoV and other bat and pangolin coronaviruses [45]. Using the same prediction algorithms, we found that miR-151a-5p, -151b (RNA hybrid MFE = −17.2 Kcal/mol) and miR-4707-3p (RNA hybrid MFE = −22 Kcal/mol) target this specific PRRA region of the S protein gene (Figure 3B). The nucleotide secondary structure of the PRRA region demonstrated that the miRNAs binding sites are accessibly located in an extended stem–loop structure of the S protein (Figure 3C) and the PRRA region is part of a stem loop in the nucleotide secondary structure of the SARS-CoV-2 S protein (Suppl. Figure S1B). The nucleotide secondary structure of the SARS-CoV genome, where PRRA region is absent, is depicted for comparison (Suppl. Figure S1A). We further assessed the three miRNAs expression in a database from primary lung fibroblasts (a cell type relatively resistant to SARS-CoV-2 infectivity) and observed high enrichment for miR-151a-5p and 151b, while miR-4707-3p was barely detectable (Suppl. Figure S1C). Interrogation of the miRNA issue ATLAS demonstrated similar enrichment of miR-151a-5p and -151b in the lungs compared to other organs, e.g., kidney, liver and lymph node, while miR-4707-3p showed similarly low expression in all organs (Suppl. Figure S1D). Collectively, these findings raise the possibility that miRNAs enriched in either SARS-CoV-2-resistant or -susceptible cell lines may provide further insights for miRNA-mediated control of SARS-CoV-2 infectivity.

### 3.4. Identification and Expression of Predicted miRNAs in SARS-CoV-2-Resistant and -Susceptible Cells

**Figure 4:**
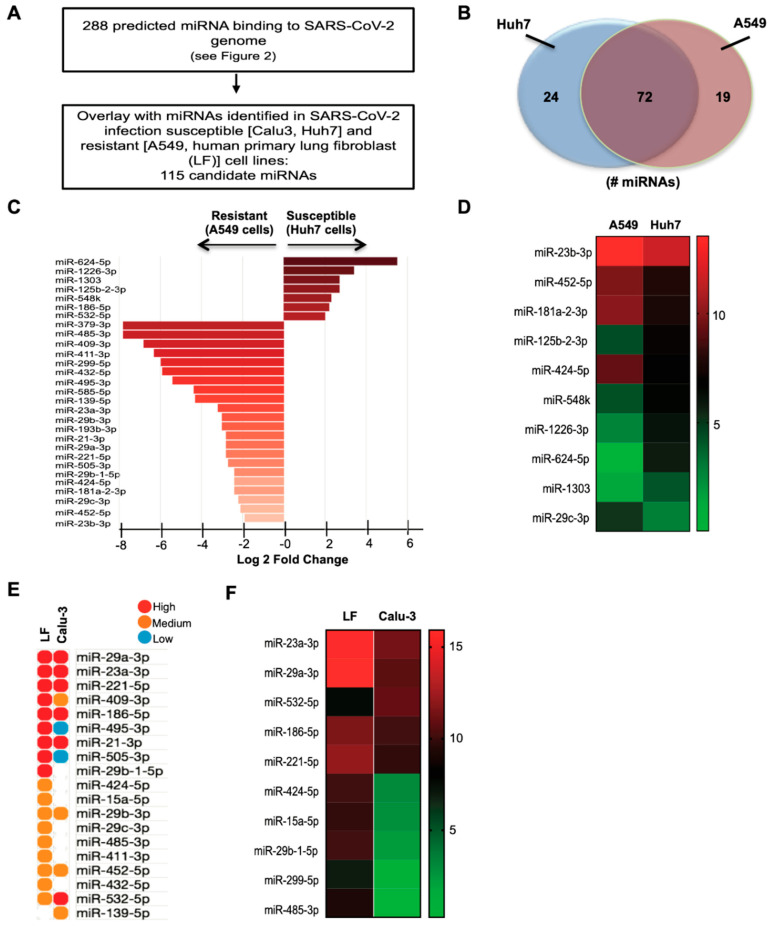
In silico analysis of predicted candidate microRNAs (miRNAs) expressed in severe acute respiratory syndrome coronavirus 2 (SARS-CoV-2)-resistant and -susceptible cell lines. (**A**) Workflow of candidate miRNAs. (**B**) Venn diagram representing the number of differentially regulated miRNAs. (**C**,**D**) Log2-transformed fold changes of most differentially regulated miRNAs (**D**) and heat map showing the top 10 differentially regulated miRNAs in A549 (resistant) and Huh7 (susceptible) cell lines. (**E**) The most differentially regulated miRNAs grouped by high (1st tercile), medium (2nd tercile), and low expression (3rd tercile) (**F**) and heat map showing the top 10 differentially regulated miRNAs expressed as log2-transformed fold changes in human primary lung fibroblasts (LF) and Calu-3 (susceptible) cells.

Here we identified several well-documented SARS-CoV-2-resistant and-susceptible cell types (Table 2) that were utilized to further investigate the expression of miRNAs predicted to bind to SARS-CoV-2 (from Figure 2) through in-silico approaches. In these cell types, ACE2/TMPRSS2 mRNA expression roughly correlates with SARS-CoV-2 infectivity. A total of 288 predicted miRNAs binding to the SARS-CoV-2 genome were overlaid on the miRNA expression profile sets of SARS-CoV-2 infection susceptible (Calu-3, Huh7) and resistant (A549, human primary lung fibroblast) cells. As a result, we identified 115 miRNAs binding to the 5′-UTR and ORF regions that were expressed in A549 (resistant) and Huh7 (susceptible) cell lines (Figure 4A,B and Suppl. Figure S2A). Specifically within the region of ORF1ab that encodes the replicase protein [46], miR-320a-3p and miR-320b are predicted to bind the *Nsp8* gene, and miR-3149 is predicted to bind to the *Nsp12* gene (Suppl. Figure S2B). MiRNA datasets for A549 and Huh7 cell lines were acquired from the same study, thereby minimizing any technical issues associated with extraction and processing. Following DESeq2 analyses of A549 and Huh7 raw datasets, 32 miRNAs were identified by differential expression analyses with an arbitrary fold change (FC) cut off of >1.9-fold and *p* < 0.05 (Figure 4C). MiR-23b-3p, miR-452-5p, and miR-181a-2-3p, the most differentially upregulated miRNAs, were enriched in the resistant A549 cells, whereas miR-624-5p, miR-1303 and miR-29c-3p were the most differentially downregulated miRNAs enriched in the susceptible Huh7 cell line (Figure 4C,D). In addition, miR-379-3p, miR-485-3p, and miR-409-3p were among the top miRNAs enriched to the resistant A549 cell line (Figure 4C). To further delineate expression of miRNAs in independent cell datasets, we extended our analyses to additional resistant (human primary lung fibroblasts) and susceptible (Calu-3) cell lines. Interestingly, 19 of the 29 miRNAs identified in A549 and Huh7 datasets were also expressed in both human primary lung fibroblasts (resistant) and Calu-3 (susceptible) cells (Figure 4E). MiR-23a-3p and miR-29a-3p were the most differentially upregulated miRNAs enriched in the resistant human lung fibroblasts, whereas miR-485-3p and miR-299-5p were amongst the top differentially downregulated miRNAs that were enriched in the resistant human lung fibroblasts (Figure 4F). Furthermore, we analyzed a recently deposited RNA-seq dataset of differentially expressed transcripts including miRNAs in Calu-3 cells after infection with SARS-CoV-2 [35]. In line with our findings in permissive and susceptible cell lines, miR-23a-3p and miR-23b-3p were significantly downregulated in Calu-3 cells infected for 24 h with SARS-CoV-2, in comparison to mock-infected cells (Table 3). Additionally, commonly enriched miRNAs between resistant and susceptible cell lines are shown in Table 4. The human tissue expression patterns of the most differentially regulated miRNAs are shown in Suppl. Figure S3.

**Figure 5:**
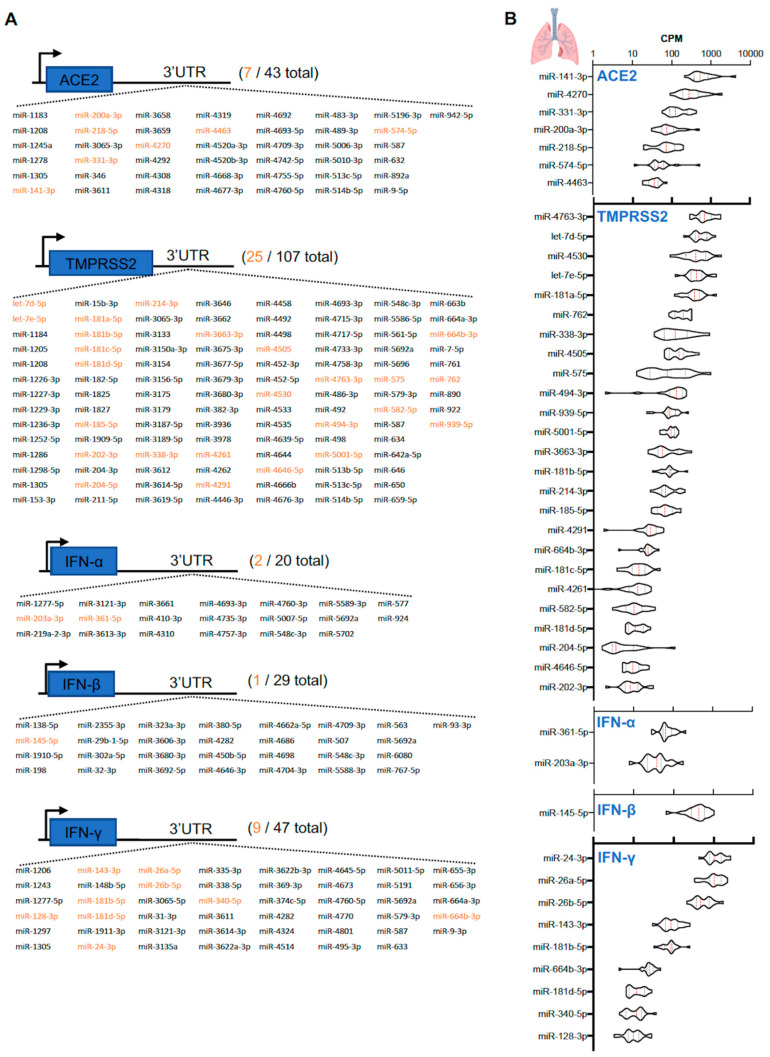
Predicted candidate microRNAs (miRNAs) targeting ACE2, TMPRSS2, interferon (IFN)-α, IFN-β, and IFN-γ mRNAs. (**A**) List of miRNAs targeting the 3′UTRs of ACE2 (total 43), TMPRSS2 (total 107), IFN-α (total 20), IFN-β (total 29), and IFN-γ mRNAs (total 47). Highlighted orange are miRNAs expressed in human lung samples [31]. (**B**) Counts per million (CPM) value of miRNAs predicted to target ACE2, TMPRSS2, IFN-α, IFN-β, and IFN-γ mRNAs found in human lungs (*n* = 18).

Given the importance of viral entry in mediating infectivity and pathogenesis of SARS-CoV-2, we explored the predicted miRNAs that target mRNAs of host receptor proteins ACE2 and TMPRSS2. In addition, we also explored miRNAs that target IFN-α, -β, and -γ as ACE2 was recently identified to be an IFN-stimulated gene [11]. Using five miRNA prediction algorithms (miRWalk, MicroT4, miRMap, RNAhybrid, and TargetScan) we identified 43 miRNAs targeting ACE2, 107 for TMPRSS2, 20 for IFN-α, 29 for IFN-β, and 47 for IFN-γ in their respective 3′UTRs (Figure 5A). Utilizing the Human MicroRNA Tissue Atlas (https://ccb-web.cs.uni-saarland.de/tissueatlas/), which contained miRNA expression profiles of 18 individual human lung tissues, we quantified the expression of these predicted miRNAs in human lungs (Figure 5B). By using a cut off of >10 CPM, the list of predicted miRNAs was reduced by more than 4-fold. There were 7 predicted lung-enriched miRNAs (the top 5 miRNAs were miR-141-3p, miR-4270, miR-331-3p, miR-200a-3p, and miR-218-5p) that targeted the ACE2 mRNA 3′-UTR and 25 predicted lung-enriched miRNAs (the top 5 miRNAs were miR-4763-3p, let-7d-5p, miR-4530, let-7e-5p, and miR-181a-5p) that targeted the TMPRSS2 mRNA 3′-UTR (Figure 5B). In addition, the 3′-UTRs of IFN-α and IFN-β mRNAs contained two (miR-203a-3p and miR-361-5p) and one (miR-145-5p) predicted binding sites, respectively, for lung-enriched miRNAs, whereas IFN-γ harbored nine (miR-128-3p, miR-143-3p, miR-181b-5p, miR-181d-5p, miR-24-3p, miR-26a-5p, miR-26b-5p, miR-340-5p, and miR-664b-3p). Notably, miR-181b-5p, miR-181d-5p, and miR-664b-3p are the only miRNAs to share both target mRNAs (TMPRSS2 and IFN-γ) in human lung tissue.

### 3.5. Identification of Predicted miRNAs to ACE2 and TMPRSS2 in SARS-CoV-2-Resistant and -Susceptible Cells

**Figure 6:**
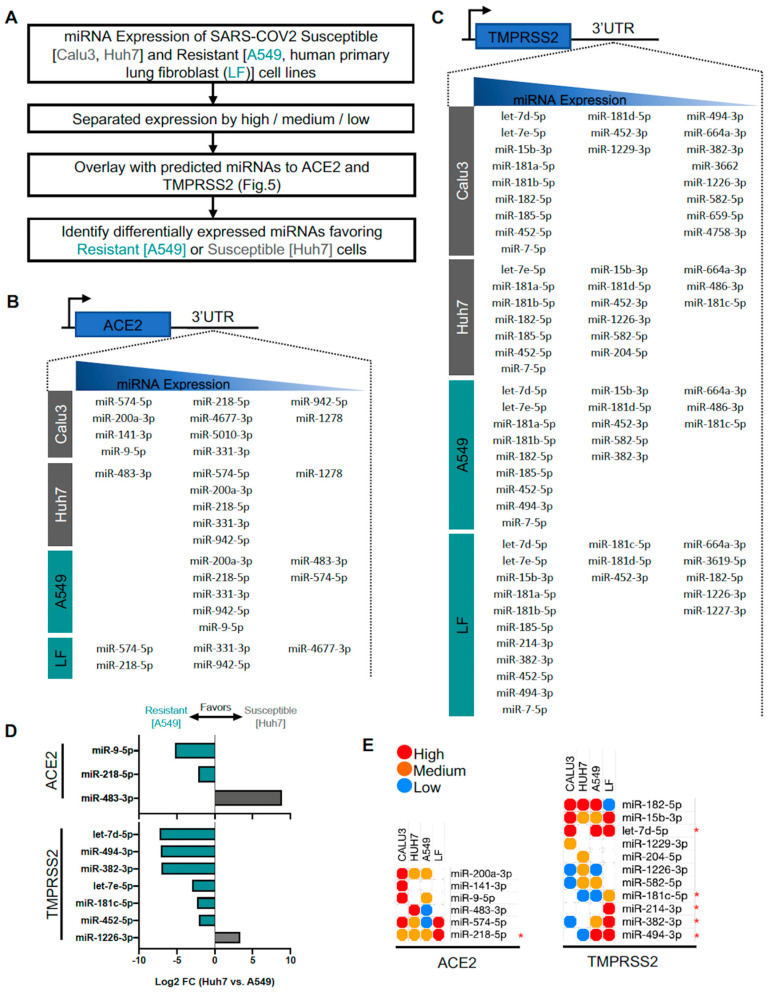
Predicted ACE2 and TMPRSS2 mRNA-targeting microRNA (miRNA) expression from severe acute respiratory syndrome coronavirus 2 (SARS-CoV-2)-resistant and -susceptible cell lines. (**A**) Workflow. (**B**,**C**) List of miRNAs grouped by high (1st tercile), medium (2nd tercile), and low expression (3rd tercile) targeting ACE2 and TMPRSS2 mRNAs, respectively. (**D**) Differential expression from Huh7 and A549 cell lines (*p* < 0.05 and >log2FC). (**E**) Heatmap of shared miRNAs between four cell lines. * identifies miRNAs more favorable to resistant cell types.

The human lung is comprised of a plethora of cell types that vary in their ability to resist infectivity of SARS-CoV-2 (Table 1). Utilizing several miRNA expression profiles of 4 different cell types (2 resistant and 2 susceptible), we applied our predicted miRNAs to identify certain miRNAs that are expressed in cells resistant to SARS-CoV-2 infectivity (Figure 6A). Similar to the human lung tissue database, we set a criterion of >10 CPM for each cell type, followed by grouping miRNAs into terciles (highest third, middle third, and lowest third). The list of predicted miRNAs for ACE2 and TMPRSS2 vary slightly between cell types (Figure 6B,C). Similar to analyses of Huh7 and A549 miRNA profiles, we performed differential expression analysis on predicted miRNAs with certain criteria (*p* < 0.05 and >log2-fold change) to identify miRNAs more unique to a particular cell type. MiR-9-5p and miR-218-5p, predicted miRNAs binding to ACE2 mRNA, are expressed higher in resistant A549 cells, while miR-483-3p is expressed higher in susceptible Huh-7 cells (Figure 6D). MiRNAs predicted to bind TMPRSS2 mRNA including let-7d-5p, miR-494-3p, miR-382-3p, let-7e-5p, miR-181c-5p, and miR-452-5p are expressed higher in A549 cells, while only miR-1226-3p is expressed higher in Huh-7 cells. These miRNAs identified to be expressed higher in A549 cells would suggest a potentially important role in providing resistance to SARS-CoV-2 infectivity by reducing ACE2 and TMPRSS2 mRNA. Furthermore, we expanded our query by incorporating Calu-3 (susceptible) and human primary lung fibroblast (LF, resistant) to identify favorable miRNAs to ACE2 and TMPRSS2 (Figure 6E). Because the miRNA expression profiles were generated by different groups, we therefore limited our analysis to semi-quantitative expression comparisons between cell types. MiRNAs that are in the same tercile group between all four cell types were excluded from the analysis (i.e., miR-452-5p and let-7e-5p targeting TMPRSS2 mRNA). Here we confirm that miR-218-5p, let-7d-5p, miR-494-3p, miR-382-3p, and miR-181c-5p are favorably expressed in resistant cells. Furthermore, we also identified that miR-214-3p targeted TMPRSS2 mRNA predominantly in resistant cells. Finally, we conducted tissue expression analyses of miRNAs targeting ACE2 and TMPRSS2 mRNAs in various human tissues including lung, kidney, lymph node, and liver (Suppl. Figure S4A).

### 3.6. Identification of Predicted miRNAs to IFN Genes in SARS-CoV-2-Resistant and -Susceptible Cells

**Figure 7:**
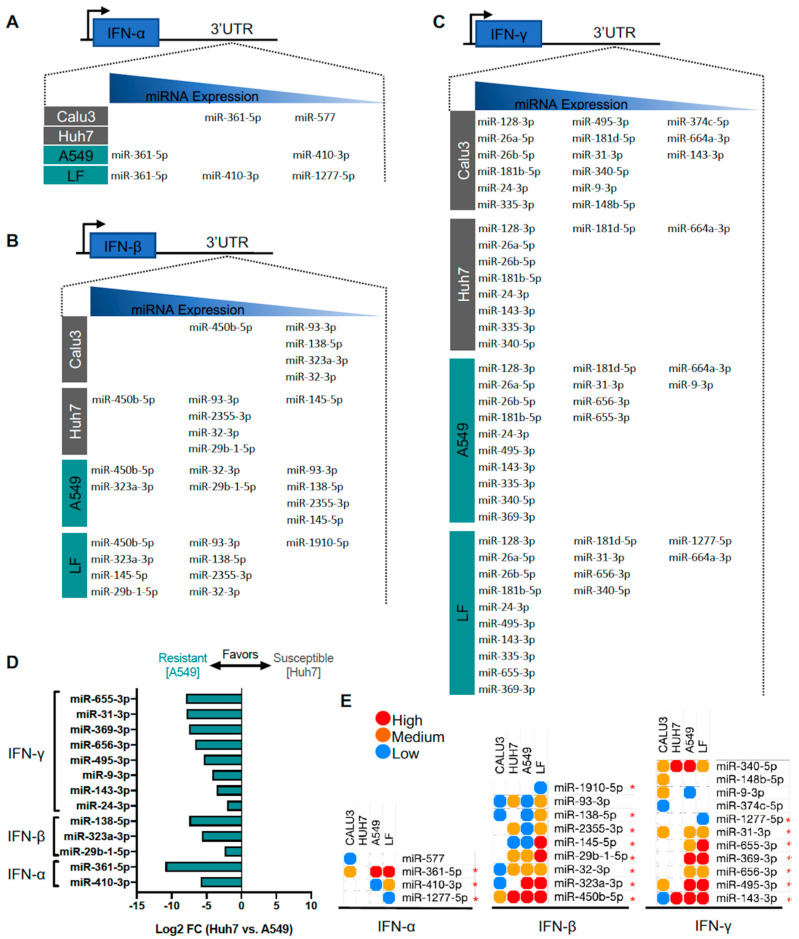
Predicted interferon (IFN)-targeting microRNA (miRNA) expression from severe acute respiratory syndrome coronavirus 2 (SARS-CoV-2)-resistant and -susceptible cell lines. (**A**–**C**) List of miRNAs grouped by high (1st tercile), medium (2nd tercile), and low expression (3rd tercile) targeting IFN-α, IFN-β, and IFN-γ, respectively. (**D**) Differential expression from Huh7 and A549 cell lines with criteria of *p* < 0.05 and >log2FC. (**E**) Heatmap of shared miRNAs between 4 cell lines. * identifies miRNAs more favorable to resistant cell types.

IFN-α and -γ are upstream drivers of ACE2 and TMPRSS2 expression [11], key proteins for SARS-CoV-2 viral entry. Similar to our efforts in finding candidate miRNAs for targeting ACE2 and TMPRSS2 mRNAs, we applied our workflow to identify miRNAs to target IFN mRNAs in resistant and susceptible cell lines (Figure 7A–C). Notably, predicted miRNAs targeting IFN-α and IFN-β mRNAs were found less in SARS-CoV-2 susceptible cells compared to resistant cell lines, suggesting there might be an overall greater amount of maintenance of these key mediators of ACE2 at the post-transcriptional level. Interestingly, when using differential expression analysis of Huh-7 and A549 cells, all the miRNAs identified are predominantly expressed in the resistant cells A549. Specifically, IFN-γ-targeting miRNAs included miR-655-3p, miR-31-3p, miR-656-3p, miR-495-3p, miR-9-3p, miR-143-3p, and miR-24-3p; IFN-β-targeting miRNAs included miR-138-5p, miR-323a-3p, and miR-9b-1-5p; and IFN-α-targeting miRNAs included miR-361-5p and miR-410-3p (Figure 7D). Furthermore, additional analysis with Calu3 (susceptible) and lung fibroblasts (resistant) cells also confirmed many of these captured miRNAs and revealed several new candidates (Figure 7E). Particularly, miR-1277-5p is shown to target both IFN-α and IFN-γ mRNAs, but was difficult to capture due to it being lowly expressed in Calu3, Huh-7, and A549 cells. Lastly, we highlight expression of IFN-α-targeting miRNAs miR-361-5p and miR-410-3p in different tissues (lung, kidney, lymph node, and liver) (Suppl. Figure S4B). Collectively, these findings highlight key miRNAs predicted to target ACE2, TMPRSS2, or IFN-α, -β, -γ mRNAs and enriched in SARS-CoV-2-resistant or -susceptible cell lines, potentially providing new insights for potentially modulating viral entry. Moreover, some of selected miRNAs targeting ACE2, TMPRSS2 and IFN mRNAs from the above analyses were dysregulated in the recent RNAseq data from lung cells infected with SARS-CoV-2. Specifically, differential expression analysis of SARS-CoV-2-infected Calu-3 cells showed upregulated expression of miR-483-3p (targeting ACE2 mRNA) and let-7d-5p (targeting TMPRSS2), while miR-450b-5p (targeting IFN-γ mRNA) expression was downregulated (Table 3).

## 4. Discussion

**Figure 8:**
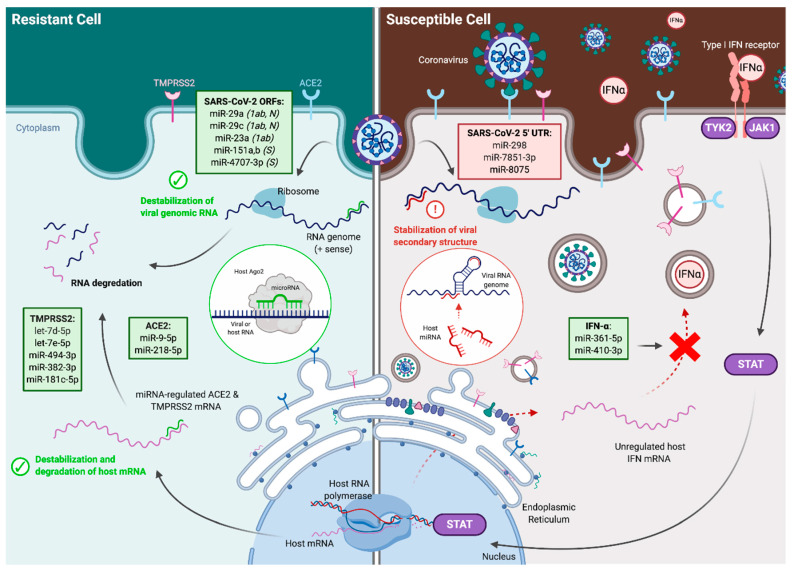
Mechanisms by which microRNAs (miRNAs) may regulate severe acute respiratory syndrome coronavirus 2 (SARS-CoV-2) infectivity. MiRNAs facilitate cellular resistance (**left**) or susceptibility (**right**) to infection. Potential regulatory mechanisms include the destabilization of viral genomic RNA; destabilization and degradation of host ACE2, TMPRSS2, and interferon (IFN) expression; or the stabilization of viral secondary structure. The specific ORFs within SARS-CoV-2 containing binding sites for miRNAs are listed in parentheses where 1ab denotes ORF1ab, N denotes nucleocapsid, and S denotes spike protein. Created using Biorender.com.

In this study, we report a collection of host miRNAs that may regulate SARS-CoV-2 infectivity in humans. We utilized validated prediction algorithms in combination with established miRNA profiling datasets to investigate potential miRNA binding to the SARS-CoV-2 RNA genome, mRNAs of viral entry proteins ACE2 and TMPRSS2, and IFN regulators implicated in driving ACE2 and TMPRSS2 expression (Figure 8). In addition, we examined differential expression of these predicted miRNAs in cells resistant or susceptible to SARS-CoV-2 infection to further deduce potential mechanisms through which cellular host miRNAs may affect SARS-CoV-2 infectivity.

### 4.1. MiRNAs Targeting the SARS-CoV-2 Viral Genome

Certain miRNAs are expressed ubiquitously, whereas others are expressed in a highly tissue-specific manner [31]. One such example is miR-122, specifically expressed in the liver, that regulates HCV replication and infectivity [50]. MiR-122 binds and stabilizes the 5′ UTR of the HCV genome, conferring resistance to enzymatic degradation and thereby contributing to increased protein production. Initial studies in different immortalized cell lines highlighted that miR-122 is highly expressed in cultured human Huh7 and mouse Hepa1-6 liver cells, but not in the human liver-derived HepG2 and cervical carcinoma-derived HeLa cells. In high correlation with miR-122 expression, HCV can only replicate in Huh7 cells, while HepG2 and HeLa are not permissive cell lines [50]. In our study, we have employed a similar approach. Previous studies established that SARS-CoV-2 exhibits varying degrees of infectivity in different commonly used cell lines [14]. To this end, we employed miRNA datasets including cell lines with documented susceptibility (Huh7 and Calu3) and resistance to SARS-CoV-2 infection (A549 and LF) in order to verify the biologic plausibility of these miRNA–target RNA interactions in vitro and predict which miRNAs may be important in regulating SARS-CoV-2 infectivity. In addition, we further analyzed available RNA-seq datasets to uncover miRNA profiling from Calu-3 cells directly infected with SARS-CoV-2 to explore whether infection resulted in altered expression profiles of the miRNAs of interest.

The single-stranded, positive-sense RNA composition of the SARS-CoV-2 genome makes it susceptible to binding and regulation by host miRNAs. Indeed, host miRNAs likely play key roles in the pathogenesis of coronaviruses across species [51]. Additionally, several miRNAs that directly target viral genomes have been described previously. For example, binding of miR-122 to HCV promotes protein translation by specifically stabilizing the secondary structure of the internal ribosomal entry site in the 5′ UTR of HCV [41]. Binding of miR-122 is necessary for viral propagation and has been successfully exploited therapeutically by the pharmaceutical miravirsen, an antisense oligonucleotide that sequesters and inhibits miR-122 to reduce HCV viral load [52,53]. Here, we have identified three miRNAs—miR-298, miR-7851-3p, and miR-8075—that are predicted to bind with high affinity to the 5′ UTR of the SARS-CoV-2 genome. Interestingly, the binding sites of all three miRNAs are within or in close proximity to stem–loop structures that are highly conserved within the *Coronaviridae* family (Figure 2D) [44]. However, it is difficult to predict in silico how endogenous miRNA binding might regulate viral genomes. For example, in contrast to miR-122 and HCV, miR-548g-3p binds to the 5′ UTR and inhibits the translation and replication of dengue virus, another positive-sense, single-strand RNA virus [42]. Similarly, other miRNAs have been shown to bind throughout the viral genome to inhibit the translation and/or promote the degradation of many other disease-causing RNA viruses including human immunodeficiency virus (HIV), hepatitis B virus, enterovirus, human T cell leukemia virus, and eastern equine encephalitis virus (EEEV) [43,54,55,56,57]. Future studies investigating the resulting changes to genomic secondary structure following miRNA binding may shed light on the potential regulation of SARS-CoV-2 by these miRNAs.

The polybasic cleavage site (RRAR) is a unique sequence in the S protein of SARS-CoV-2, at the junction of S1 and S2, the two subunits of the spike protein and absent in SARS-CoV and other bat and pangolin coronaviruses. A leading proline results in the addition of O-linked glycans to S673, T678 and S686, which flank the cleavage site (PRRA) (Figure 3B) [25,58]. As previously suggested, this allows effective cleavage by furin and other proteases and has a role in determining viral infectivity [59]. We show that miR-151a-5p, -151b and miR-4707-3p may target the novel PRRA region in the SARS-CoV-2 genome, and assessment of the secondary structure in this region supports accessibility for miRNAs binding. The enriched expression of miR-151-5p and 151b in the lungs and lung primary fibroblasts suggests a potential implication of these miRNAs in the spike protein stability and SARS-CoV-2 RNA stability. Additionally, Nsp7, Nsp8, and Nsp12 have also recently been identified as important proteins in viral replication and are targeted by the investigational therapeutic remdesivir [60,61]. Here, we also show that miRs-320a-3p, -320b and -3149 were predicted to bind to the Nsp8 and Nsp12 genomic regions of the replicase polyprotein 1ab (PP1ab), a region that is important in transcription and replication of viral RNAs [46]. MiR-320 family was implicated in a number of viral infections. MiR-320a-3p was shown to inhibit mink enteritis virus (MEV) replication [62], whereas miR-320b was significantly increased in human plasma samples following Ebola infection [63]. The relevance and applicability of these findings to SARS-CoV-2 infection needs to be further delineated.

RNA viruses can quickly mutate their genomes in response to evolutionary pressure due to the lack of proofreading activity from virally-encoded polymerases [64]. MiRNA binding sites, particularly the 6 to 8 nt seed regions, are therefore likely evolutionarily conserved due to strong positive selection. As noted previously, it is difficult to predict how miRNA binding may regulate viral translation or replication in vivo. Moreover, even miRNAs that negatively regulate genomic stability and limit viral replication may paradoxically result in increased viral infectivity at the organismal level due to cell type-specific miRNA expression. For example, miR-142-3p is exclusively expressed in hematopoietic cells and is necessary for proper hematopoietic cell differentiation [65,66]. Eastern Equine Encephalitis virus (EEEV), another single-strand, positive sense RNA virus, contains three highly-conserved miR-142-3p binding sites in its 3′ UTR, and viral replication is potently inhibited in cells expressing miR-142-3p [43]. Trobaugh et al. demonstrated that repression of EEEV replication in myeloid cells is alleviated upon deletion of miR-142-3p binding sites, and infected myeloid cells contribute to potent type I IFN response [43]. However, despite increased viral replication in myeloid cells and lymph nodes, disease severity was reduced in mice infected with EEEV containing deleted binding sites due in part to the limited capacity for activation of the innate immune system. In this way, there is a strong positive selective pressure for the maintenance of miR-142-3p binding sites in the 3′ UTR of EEEV due to cell-specific miRNA expression. Interestingly, in addition to miR-142-3p, miR-29a has also been identified as a critical regulator of hematopoietic cell differentiation, and loss of hematopoietic cell miR-29a expression has been associated with the development of myelodysplastic syndromes and leukemias [65,67,68]. We also predict that many other potential miRNAs target the 5′ UTR, coding regions, and intergenic non-coding regions of the SARS-CoV-2 genome (Figure 2), and cell-specific expression of these miRNAs may considerably impact the regulatory control over SARS-CoV-2 replication and infectivity on both the cellular and organismal level.

Several independent analyses of single-cell RNA-sequencing datasets demonstrated that ACE2 and TMPRSS2 are co-expressed in only a limited subset of cell types, primarily secretory cells of the upper and middle airways [11,12,13]. Because SARS-CoV-2 is an enveloped virus, it does not require host-cell lysis for viral release, leading to speculation that SARS-CoV-2 preferentially infects secretory cell types to promote the rapid release of viral particles. In this context, viral mechanisms that promote host-cell survival would likely be advantageous in enhancing viral replication and propagation. MiR-29 has recently emerged as an important regulator of cell survival and apoptosis by targeting several components of pathways critical to cell proliferation and survival including p53 inhibitors *p85α* and *CDC42* as well as *Bcl2*, *CCND2*, and *c-Myc* [69,70,71]. Similarly, miR-15a -15b, and miR-16 have been shown to play critical roles in these processes by targeting a similar cassette of genes such as *Bcl2*, *CCND1*, *CCNE*, and *Mcl1* [72,73,74], and miR-23b regulates lungs cells proliferation by targeting Cyclin G1 (*CCNG1*) [75]. Here, we show that miR-29a, -15a, b, -16, and -23a,b have several high-probability predicted binding sites throughout the SARS-CoV-2 genome and exhibited high expression in human lungs (Figure 2 and Figure 3). By binding these miRNAs at multiple high-affinity sites, SARS-CoV-2 may functionally sequester their cytosolic pool [76] and promote the survival of infected cells through the de-repression of cell cycle and apoptosis target genes. Data from SARS-CoV-2-infected Calu-3 cells may support this paradigm, demonstrating a decrease in miR-23a and miR-23b following SARS-CoV-2 infection (Table 3).

We also show that several miRNAs may directly play a role in regulating viral infectivity using established miRNA profiling datasets of both Huh7 (high SARS-CoV-2 infectivity) and A549 (low SARS-CoV-2 infectivity). Our correlative results suggest that several miRNAs may be of potential interest as therapeutic targets. For example, miR-23a, -23b, miR-29a, -29c (-3p) and -29b-1-5p are predicted to bind several regions of the SARS-CoV-2 genome, including the regions encoding ORF1ab (miR-23a and miR-29a, -29-c, -29b-1), the Nucleocapsid (miR-29a, -29c) proteins (Figure 2). At the same time, we observed that miR-29a, -29c and miR-23a, -23b are highly expressed in the cell lines that show low SARS-CoV-2 infectivity (A549 and primary lung fibroblasts), while showing much lower expression in cell lines with high SARS-CoV-2 infectivity (Calu-3 and Huh7; Figure 4). As previously observed, miRNA binding to different genomic regions that code for viral proteins can interfere with the viral replication, translation or infectivity [19]. Whether the increased expression of miR-23a and miR-29a, -29c in non-permissive cell lines may play a role in SARS-CoV-2 infectivity needs to be further assessed by miRNA loss- and gain-of function studies in respective cell lines. Moreover, the two miRNA families (-23 and -29) are highly expressed in healthy lungs (Figure 3). Future analysis of these miRNAs in the lung tissues or plasma of COVID-19 infected patients will be informative for their potential regulation and correlation with increasing severity of disease.

Our study builds upon previous computational analyses that predict interactions between host miRNAs and SARS-CoV-2. Similar to our analytic strategy, Fulzele et al. recently utilized miRDB (a cut off of target score ≥95) to predict miRNAs that bind to the SARS-CoV-2 genome [77]. Because miRNA target prediction algorithms are not specifically designed to predict miRNA-viral genome interactions, we utilized a more liberal cut off of target score ≥70 along with verification by a second prediction tool, RNAhybrid. Using this strategy, we identify many of the same miRNA targets such as miR-15a-5p and miR-15b-5p in addition to a host of other potentially pathogenically important host miRNAs. Chow and Salmena utilized RNA22 to predict miRNAs that may bind the SARS-CoV-2 genome and analyzed their expression in SARS-CoV-2-infected versus uninfected Calu3 cells [78]. By utilizing more robust miRNA-binding prediction methods through two independent prediction tools (miRDB and RNAhybrid), our analysis builds on that of Chow and Salmena and provides additional candidate miRNAs for future in vitro and in vivo investigation.

In addition to potential regulation by host miRNAs, SARS-CoV-2 may encode viral miRNAs or other non-coding RNA transcripts that impact its infectivity [79,80]. For example, SARS-CoV encodes several small viral RNAs 18–22 nt capable of silencing target mRNA expression through non-canonical miRNA regulatory pathways [81]. Treatment of SARS-CoV-infected mice with small viral RNA inhibitors resulted in reduced infection severity, lower levels of inflammation, and higher mRNA expression of antiviral proteins. To date, there have been no confirmed SARS-CoV-2-encoded miRNAs, although several studies predict the existence of such virally-encoded miRNAs [82,83]. It will be important to determine in future studies whether SARS-CoV-2 encodes miRNAs capable of altering viral infectivity and whether they are candidates for therapeutic intervention.

### 4.2. MiRNAs Targeting Viral Entry Proteins and IFNs

Ziegler et al. [11] recently established ACE2 as an IFN-stimulated gene (ISG), primarily in response to IFN-α stimulation [11]. We therefore investigated potential miRNA regulators of IFN-α, -β, and -γ that may play indirect roles in susceptibility to SARS-CoV-2 infection (Figure 5 and Figure 7). Strikingly, all miRNAs predicted to bind the 3′ UTR of IFN genes are more highly expressed in cell lines resistant to SARS-CoV-2 infection. We found three miRNAs that were co-expressed in both the SARS-CoV-2-resistant cell line A549 and human lung tissue: miR-361-5p targeting IFN-α mRNA and miR-24-3p and miR-143-3p targeting IFN-γ mRNA. In qualitative analyses including additional Calu3 and LF cell lines, miR-361-5p maintained a consistent pattern of high expression in those resistant to SARS-CoV-2 infection and low expression in those permissive to infection, making this an intriguing target for further study. Furthermore, miR-495-3p, which targets the ORF1ab region of the SARS-CoV-2 genome and is highly expressed the resistant cell lines A549 and human primary lung fibroblasts, is also predicted to target IFN-γ 3′-UTR. MiR-495-3p expression was significantly upregulated in mouse lungs infected with H5N2 virus [84] and in human serum following Hepatitis C infection [85]. Whether such upregulation is also valid in response to SARS-CoV-2 infection as a possible protective mechanism to inhibit ACE2 induction via IFN-γ remains to be determined in subsequent in vitro and in vivo studies.

Importantly, dedicated in vitro and in vivo studies demonstrated that ACE2 induction by IFN was considerably weaker in immortalized cell lines and absent in mouse primary tracheal epithelial cells, suggesting a substantial degree of cell and species specificity to this previously unrecognized regulation of SARS-CoV-2 entry factor ACE2 [11]. We have identified several candidate miRNAs that may offer an additional level of regulation of ACE2 with a similar degree of cell specificity. Using several established bioinformatic miRNA target prediction tools, we provide a collection of miRNAs that may target mRNAs of SARS-CoV-2 entry factors ACE2 and TMPRSS2 as well as upstream regulators IFNs with high-probability (Figure 5A). By evaluating the expression of these miRNAs in RNA-sequencing datasets of human lung tissue, we predict 7 miRNAs targeting ACE2 mRNA (e.g., the top 3 are miR-141-3p, miR-4270, miR-331-3p) and 25 miRNAs targeting TMPRSS2 mRNA (e.g., the top 9 are miR-4763-3p, let-7d-5p, miR-4530, let-7e-5p, miR-181a-5p, miR-762, miR-338-3p, miR-4505, miR-575) to be abundant in human lung tissue, establishing biologic plausibility for these miRNA–mRNA interactions (Figure 5B). 

We found ten total miRNA—three targeting ACE2 mRNA (miR-9-5p, miR-218-5p, and miR-483-3p) and seven targeting TMPRSS2 mRNA (let-7d-5p, miR-494-3p, miR-382-3p, let-7e-5p, miR-181c-5p, miR-452-5p, and miR-1226-3p)—that demonstrated statistically significant differential expression favoring either resistance (i.e., higher expression in A549) or susceptibility to SARS-CoV-2 infection (i.e., higher expression in Huh7; Figure 6D). We also found that of these eight, miR-218-5p (targeting ACE2 mRNA), miR-382-3p, miR-494-3p, let-7d-5p, let-7e-5p, and miR-181c-5p (targeting TMPRSS2 mRNA) are also expressed in human lung tissue. Importantly, miR 382-3p and miR 494-3p also demonstrated higher expression in SARS-CoV-2-resistant LF cells. Additionally, both miR-218 and let-7d were previously shown to be expressed in bronchoalveolar stem cells, which are susceptible to SARS-CoV expression in part due to surface ACE2 expression [86,87]. Further dedicated miRNA profiling and target gene expression analyses will be insightful regarding the impact of these miRNAs on the relative susceptibility or resistance to infection in cells expressing these miRNAs.

Using RNAseq data from Calu-3 cells infected with SARS-CoV-2, we identified differentially expressed miRNAs targeting the genes involved in virus entry. Interestingly, several miRNAs targeting mRNAs of viral entry proteins were upregulated following infection such as miR-483-3p targeting ACE2 mRNA and miR-181c-5p and let-7d-5p targeting TMPRS2 mRNA. Although we found that miR-483-3p is expressed higher in cells susceptible to SARS-CoV-2 infection, its upregulation (4.46-fold) along with the upregulation of miR-181c-5p and let-7d-5p after infection in Calu-3 cells may represent compensatory responses of host cells to inhibit SARS-CoV-2 entry by targeting ACE2 and TMPRSS2 mRNA. Indeed, ACE2 expression levels were found downregulated in mouse lungs after SARS-CoV infection [88].

## 5. Conclusions

Overall, in this study, we establish a framework for future investigation of miRNAs regulating SARS-CoV-2 infectivity in human cells. Leveraging miRNA profiling from cells resistant or susceptible to SARS-CoV-2 infection, we provide a preliminary resource of high-probability miRNAs governing SARS-CoV-2 infectivity through two potential mechanisms: (1) the direct binding of host miRNAs to the SARS-CoV-2 genome, which have the potential to both promote and prevent SARS-CoV-2 infection; and (2) regulation of the mRNAs of viral entry proteins ACE2 and TMPRSS2 including regulation via their upstream IFN modulators. Further studies are needed to validate potential miRNA binding sites in both SARS-CoV-2 and mRNAs of entry proteins. Similar to other host miRNA–virus interactions, these miRNAs may represent targets for potential therapeutic intervention in COVID-19 patients.

## Figures and Tables

**Table 1:** Comparison of select genomic regions between severe acute respiratory syndrome coronavirus 2 (SARS-CoV-2) reference sequence and the five largest outliers found during BLAST sequence identity analysis.

Location	Wuhan, China (RefSeq)	Washington, USA	Washington, USA	California, USA	Washington, USA	Punjab, Pakistan
**GenBank**	MN908947.3	MT246485.1	MT345855.1	MT258382.1	MT293170.1	MT262993.1
**Overall**						
Bases	29,903	29,775	29,859	29,885	29,798	29,836
Sequence identity ^a^	-	99.85% ^b^	99.85% ^b^	99.87% ^b^	99.88% ^b^	99.88%
**5′ UTR**						
Bases	265	137	234	247	151	265
Sequence identity ^a^	-	82.5% ^b^	91.45% ^b^	99.60	92.72% ^b^	100%
Mutations	-	-	C→T	C→T	-	-
**S protein**						
Bases	3822	3822	3822	3822	3822	3822
Sequence identity ^a^	-	100%	100%	99.95%	100%	100%
Mutations	-	-	-	T→W (either T or A); A→G	-	-
**3′ UTR**						
Bases	229	229	198	229	238	198
Sequence identity ^a^	-	98.69%	100%	93.45% ^b^	97.38% ^b^	100%
Mutations	-	TGAC → AAAA	-	G→R (either G or A); C→M (either C or A)	9 nt insertion in poly-A tail	-

^a^ Sequence identity was based on BLAST analyses with Wuhan-Hu-1 (GenBank accession: MN908947.3) as a reference sequence. ^b^ NCBI sequence contains short “N” repeat (unknown sequence) due to unreliable sequencing, accounting for a large proportion of sequence deviation from reference sequence.

**Table 2:** ACE2 and TMPRSS2 expression and reported severe acute respiratory syndrome coronavirus 2 (SARS-CoV-2) infectivity in various liver and lung cells.

	Source	ACE2/TMPRSS2 Expression	SARS-CoV-2 Infectivity
Huh7 [8,14,32,33]	Liver	High [47]	Moderate
A549 [8,14,32,33]	Lung	Variable [48]	Low to Moderate
Calu-3 [8,14,32,34]	Lung	High [49]	High
Primary human lung fibroblasts [12,13]	Lung	None	Low

Predicted miRNAs that target host genes associated with the pathogenesis of SARS-CoV-2.

**Table 3:** Differential expression of candidate microRNAs (miRNAs) in severe acute respiratory syndrome coronavirus 2 (SARS-CoV-2)-infected (24 h) vs. non-infected Calu-3 cells from Wyler et al. [35].

miRNA	Log2-Fold Change	*p*-value	Favors ^a^
**ORF1ab**			
miR-139-5p	0.945	0.0305	Resistant
miR-664b-3p	0.733	0.0206	-
miR-23a-3p	−0.761	0.0015	Resistant
miR-548d-3p	−0.807	0.0517	-
miR-15b-5p	−0.812	0.0001	-
miR-23c	−0.910	0.0049	-
miR-23b-3p	−0.991	<0.0001	Resistant
miR-374b-3p	−1.069	0.0217	-
miR-374a-3p	−1.582	0.0001	-
**Nucleocapsid**			
miR-103a-3p	0.904	0.0005	-
miR-107	1.040	0.0003	-
**ACE2**			
miR-483-3p	4.460	<0.0001	Susceptible
miR-4463	3.048	<0.0001	-
**TMPRSS2**			
miR-181c-5p	0.892	0.0134	Resistant
miR-664b-3p	0.733	0.0206	-
miR-182-5p	0.704	0.0134	Susceptible
let-7d-5p	0.464	0.0053	Resistant
miR-181a-5p	0.419	0.0422	-
miR-15b-3p	−0.810	0.0008	-
miR-494-3p	−0.961	0.0326	Resistant
**IFN-β**			
miR-450b-5p	−1.441	0.0020	Resistant
**IFN-γ**			
miR-664b-3p	0.733	0.0206	-
miR-26b-5p	−0.475	0.0313	-
miR-374c-5p	−1.228	0.0096	-

^a^ Susceptible and resistant refers to cell line analyses as per Figures 4, 6, or 7.

**Table 4:** Concordant microRNA expression between A549 and Huh7 cells.

	log2FC	p_adj_
miR-140-3p	−0.007953398	0.974817048
miR-32-3p	0.013920456	0.974817048
miR-374a-5p	0.020538776	0.945449302
miR-196b-5p	0.025080141	0.946019864
miR-339-5p	0.029116789	0.949059007
miR-3158-3p	0.029234925	0.97722196
miR-320c	0.033177006	0.970481801
miR-423-3p	0.039739622	0.860137595
miR-331-5p	0.04015155	0.957965014
miR-1180-3p	0.042520619	0.897231993
miR-1229-3p	0.049456491	0.979826711
miR-6803-3p	0.069110586	0.956513365
miR-339-3p	0.075904611	0.883114045
miR-4326	0.149533463	0.815150225
miR-16-2-3p	0.157983774	0.530911494
miR-6087	0.178211797	0.945394126
miR-25-5p	0.181172206	0.692047229
miR-148b-3p	0.20381122	0.369688028
miR-126-3p	0.20432634	0.517498738
miR-760	0.204547203	0.876649033
miR-200b-5p	0.206829005	0.813533675
miR-1307-5p	0.221466282	0.90711043
miR-454-3p	0.226527458	0.372951464
miR-107	0.232189082	0.645482953
miR-301a-3p	0.234614774	0.88095324
miR-200b-3p	0.241301831	0.267692191
miR-324-3p	0.254720466	0.852842736

## Supplementary Materials

The following are available online at https://www.mdpi.com/2073-4425/11/11/1354/s1, Figure S1: Secondary structure of SARS-CoV and SARS-CoV-2 spike proteins and lung expression of miRNA binding in the PRRA region of the SARS-CoV-2 spike protein, Figure S2: Differential expression of miRNAs binding the SARS-CoV-2 genome between resistant versus susceptible cell lines. Figure S3: Expression of the most differentially regulated miRNAs in relevant human tissues, Figure S4: Predicted miRNAs of host genes expression across tissues.

## References

[1] World Health Organization Coronavirus Disease (COVID-19) Pandemic. https://www.who.int/emergencies/diseases/novel-coronavirus-2019.

[2] Wang C., Horby P.W., Hayden F.G., Gao G.F. (2020). A novel coronavirus outbreak of global health concern. Lancet.

[3] Zhu N., Zhang D., Wang W., Li X., Yang B., Song J., Zhao X., Huang B., Shi W., Lu R. (2020). A novel coronavirus from patients with pneumonia in China, 2019. N. Engl. J. Med..

[4] Huang C., Wang Y., Li X., Ren L., Zhao J., Hu Y., Zhang L., Fan G., Xu J., Gu X. (2020). Clinical features of patients infected with 2019 novel coronavirus in Wuhan, China. Lancet.

[5] Guan W.-J., Ni Z.-Y., Hu Y., Liang W.-H., Ou C.-Q., He J.-X., Liu L., Shan H., Lei C.-l., Hui D.S. (2020). Clinical characteristics of coronavirus disease 2019 in China. N. Engl. J. Med..

[6] Lu R., Zhao X., Li J., Niu P., Yang B., Wu H., Wang W., Song H., Huang B., Zhu N. (2020). Genomic characterisation and epidemiology of 2019 novel coronavirus: Implications for virus origins and receptor binding. Lancet.

[7] Yan R., Zhang Y., Li Y., Xia L., Guo Y., Zhou Q. (2020). Structural basis for the recognition of SARS-CoV-2 by full-length human ACE2. Science.

[8] Hoffmann M., Kleine-Weber H., Schroeder S., Krüger N., Herrler T., Erichsen S., Schiergens T.S., Herrler G., Wu N.-H., Nitsche A. (2020). SARS-CoV-2 cell entry depends on ACE2 and TMPRSS2 and is blocked by a clinically proven protease inhibitor. Cell.

[9] Wang Q., Zhang Y., Wu L., Niu S., Song C., Zhang Z., Lu G., Qiao C., Hu Y., Yuen K.-Y. (2020). Structural and functional basis of SARS-CoV-2 entry by using human ACE2. Cell.

[10] Shang J., Ye G., Shi K., Wan Y., Luo C., Aihara H., Geng Q., Auerbach A., Li F. (2020). Structural basis of receptor recognition by SARS-CoV-2. Nature.

[11] Ziegler C., Allon S.J., Nyquist S.K., Mbano I., Miao V.N., Cao Y., Yousif A.S., Bals J., Hauser B.M., Feldman J. (2020). SARS-CoV-2 receptor ACE2 is an interferon-stimulated gene in human airway epithelial cells and is enriched in specific cell subsets across tissues. Cell.

[12] Sungnak W., Huang N., Bécavin C., Berg M., Queen R., Litvinukova M., Talavera-López C., Maatz H., Reichart D., Sampaziotis F. (2020). SARS-CoV-2 entry factors are highly expressed in nasal epithelial cells together with innate immune genes. Nat. Med..

[13] Lukassen S., Chua R.L., Trefzer T., Kahn N.C., Schneider M.A., Muley T., Winter H., Meister M., Veith C., Boots A.W. (2020). SARS-CoV-2 receptor ACE2 and TMPRSS2 are primarily expressed in bronchial transient secretory cells. EMBO J..

[14] Chu H., Chan J., Yuen T., Shuai H., Yuan S., Wang Y., Hu B., Yip C., Tsang J., Huang X. (2020). Comparative tropism, replication kinetics, and cell damage profiling of SARS-CoV-2 and SARS-CoV with implications for clinical manifestations, transmissibility, and laboratory studies of COVID-19: An observational study. Lancet Microbe.

[15] Gebert L.F., MacRae I.J. (2019). Regulation of microRNA function in animals. Nat. Rev. Mol. Cell Biol..

[16] Kincaid R.P., Sullivan C.S. (2012). Virus-encoded microRNAs: An overview and a look to the future. PLoS Pathog..

[17] Dölken L., Krmpotic A., Kothe S., Tuddenham L., Tanguy M., Marcinowski L., Ruzsics Z., Elefant N., Altuvia Y., Margalit H. (2010). Cytomegalovirus microRNAs facilitate persistent virus infection in salivary glands. PLoS Pathog..

[18] Bauman Y., Nachmani D., Vitenshtein A., Tsukerman P., Drayman N., Stern-Ginossar N., Lankry D., Gruda R., Mandelboim O. (2011). An identical miRNA of the human JC and BK polyoma viruses targets the stress-induced ligand ULBP3 to escape immune elimination. Cell Host Microbe.

[19] Trobaugh D.W., Klimstra W.B. (2017). MicroRNA regulation of RNA virus replication and pathogenesis. Trends Mol. Med..

[20] Edgar R.C. (2004). MUSCLE: Multiple sequence alignment with high accuracy and high throughput. Nucleic Acids Res..

[21] Hatcher E.L., Zhdanov S.A., Bao Y., Blinkova O., Nawrocki E.P., Ostapchuck Y., Schäffer A.A., Brister J.R. (2017). Virus Variation Resource–improved response to emergent viral outbreaks. Nucleic Acids Res..

[22] Letunic I., Bork P. (2019). Interactive Tree Of Life (iTOL) v4: Recent updates and new developments. Nucleic Acids Res..

[23] Altschul S.F., Gish W., Miller W., Myers E.W., Lipman D.J. (1990). Basic local alignment search tool. J. Mol. Biol..

[24] Zhang T., Wu Q., Zhang Z. (2020). Probable pangolin origin of SARS-CoV-2 associated with the COVID-19 outbreak. Curr. Biol..

[25] Andersen K.G., Rambaut A., Lipkin W.I., Holmes E.C., Garry R.F. (2020). The proximal origin of SARS-CoV-2. Nat. Med..

[26] Chen Y., Wang X. (2020). miRDB: An online database for prediction of functional microRNA targets. Nucleic Acids Res..

[27] Gupta A., Nagilla P., Le H.-S., Bunney C., Zych C., Thalamuthu A., Bar-Joseph Z., Mathavan S., Ayyavoo V. (2011). Comparative expression profile of miRNA and mRNA in primary peripheral blood mononuclear cells infected with human immunodeficiency virus (HIV-1). PLoS ONE.

[28] Krüger J., Rehmsmeier M. (2006). RNAhybrid: microRNA target prediction easy, fast and flexible. Nucleic Acids Res..

[29] Zuker M., Stiegler P. (1981). Optimal computer folding of large RNA sequences using thermodynamics and auxiliary information. Nucleic Acids Res..

[30] Sticht C., De La Torre C., Parveen A., Gretz N. (2018). miRWalk: An online resource for prediction of microRNA binding sites. PLoS ONE.

[31] Ludwig N., Leidinger P., Becker K., Backes C., Fehlmann T., Pallasch C., Rheinheimer S., Meder B., Stähler C., Meese E. (2016). Distribution of miRNA expression across human tissues. Nucleic Acids Res..

[32] Ou X., Liu Y., Lei X., Li P., Mi D., Ren L., Guo L., Guo R., Chen T., Hu J. (2020). Characterization of spike glycoprotein of SARS-CoV-2 on virus entry and its immune cross-reactivity with SARS-CoV. Nat. Commun..

[33] Harcourt J., Tamin A., Lu X., Kamili S., Sakthivel S.K., Murray J., Queen K., Tao Y., Paden C.R., Zhang J. (2020). Severe Acute Respiratory Syndrome Coronavirus 2 from Patient with 2019 Novel Coronavirus Disease, United States. Emerg. Infect. Dis..

[34] Sheahan T.P., Sims A.C., Zhou S., Graham R.L., Pruijssers A.J., Agostini M.L., Leist S.R., Schäfer A., Dinnon K.H., Stevens L.J. (2020). An orally bioavailable broad-spectrum antiviral inhibits SARS-CoV-2 in human airway epithelial cell cultures and multiple coronaviruses in mice. Sci. Transl. Med..

[35] Wyler E., Mösbauer K., Franke V., Diag A., Gottula L.T., Arsie R., Klironomos F., Koppstein D., Ayoub S., Buccitelli C. (2020). Bulk and single-cell gene expression profiling of SARS-CoV-2 infected human cell lines identifies molecular targets for therapeutic intervention. bioRxiv.

[36] Müller S., Glaß M., Singh A.K., Haase J., Bley N., Fuchs T., Lederer M., Dahl A., Huang H., Chen J. (2019). IGF2BP1 promotes SRF-dependent transcription in cancer in a m6A-and miRNA-dependent manner. Nucleic Acids Res..

[37] Love M.I., Huber W., Anders S. (2014). Moderated estimation of fold change and dispersion for RNA-seq data with DESeq2. Genome Biol..

[38] Zhang X., Chu H., Wen L., Shuai H., Yang D., Wang Y., Hou Y., Zhu Z., Yuan S., Yin F. (2020). Competing endogenous RNA network profiling reveals novel host dependency factors required for MERS-CoV propagation. Emerg. Microbes Infect..

[39] Wei P., Xie Y., Abel P.W., Huang Y., Ma Q., Li L., Hao J., Wolff D.W., Wei T., Tu Y. (2019). Transforming growth factor (TGF)-β1-induced miR-133a inhibits myofibroblast differentiation and pulmonary fibrosis. Cell Death Dis..

[40] Fukuhara T., Kambara H., Shiokawa M., Ono C., Katoh H., Morita E., Okuzaki D., Maehara Y., Koike K., Matsuura Y. (2012). Expression of microRNA miR-122 facilitates an efficient replication in nonhepatic cells upon infection with hepatitis C virus. J. Virol..

[41] Schult P., Roth H., Adams R.L., Mas C., Imbert L., Orlik C., Ruggieri A., Pyle A.M., Lohmann V. (2018). microRNA-122 amplifies hepatitis C virus translation by shaping the structure of the internal ribosomal entry site. Nat. Commun..

[42] Wen W., He Z., Jing Q., Hu Y., Lin C., Zhou R., Wang X., Su Y., Yuan J., Chen Z. (2015). Cellular microRNA-miR-548g-3p modulates the replication of dengue virus. J. Infect..

[43] Trobaugh D.W., Gardner C.L., Sun C., Haddow A.D., Wang E., Chapnik E., Mildner A., Weaver S.C., Ryman K.D., Klimstra W.B. (2014). RNA viruses can hijack vertebrate microRNAs to suppress innate immunity. Nature.

[44] Yang D., Leibowitz J.L. (2015). The structure and functions of coronavirus genomic 3′ and 5′ ends. Virus Res..

[45] Walls A.C., Park Y.-J., Tortorici M.A., Wall A., McGuire A.T., Veesler D. (2020). Structure, function, and antigenicity of the SARS-CoV-2 spike glycoprotein. Cell.

[46] Wu C., Liu Y., Yang Y., Zhang P., Zhong W., Wang Y., Wang Q., Xu Y., Li M., Li X. (2020). Analysis of therapeutic targets for SARS-CoV-2 and discovery of potential drugs by computational methods. Acta Pharm. Sin. B.

[47] Lambert D.W., Yarski M., Warner F.J., Thornhill P., Parkin E.T., Smith A.I., Hooper N.M., Turner A.J. (2005). Tumor necrosis factor-α convertase (ADAM17) mediates regulated ectodomain shedding of the severe-acute respiratory syndrome-coronavirus (SARS-CoV) receptor, angiotensin-converting enzyme-2 (ACE2). J. Biol. Chem..

[48] Jia H.P., Look D.C., Shi L., Hickey M., Pewe L., Netland J., Farzan M., Wohlford-Lenane C., Perlman S., McCray P.B. (2005). ACE2 receptor expression and severe acute respiratory syndrome coronavirus infection depend on differentiation of human airway epithelia. J. Virol..

[49] Tseng C.-T.K., Tseng J., Perrone L., Worthy M., Popov V., Peters C.J. (2005). Apical entry and release of severe acute respiratory syndrome-associated coronavirus in polarized Calu-3 lung epithelial cells. J. Virol..

[50] Jopling C.L., Yi M., Lancaster A.M., Lemon S.M., Sarnow P. (2005). Modulation of hepatitis C virus RNA abundance by a liver-specific MicroRNA. Science.

[51] Liu X., Zhu L., Liao S., Xu Z., Zhou Y. (2015). The porcine microRNA transcriptome response to transmissible gastroenteritis virus infection. PLoS ONE.

[52] Janssen H.L., Reesink H.W., Lawitz E.J., Zeuzem S., Rodriguez-Torres M., Patel K., van der Meer A.J., Patick A.K., Chen A., Zhou Y. (2013). Treatment of HCV infection by targeting microRNA. N. Engl. J. Med..

[53] Lanford R.E., Hildebrandt-Eriksen E.S., Petri A., Persson R., Lindow M., Munk M.E., Kauppinen S., Ørum H. (2010). Therapeutic silencing of microRNA-122 in primates with chronic hepatitis C virus infection. Science.

[54] Huang J., Wang F., Argyris E., Chen K., Liang Z., Tian H., Huang W., Squires K., Verlinghieri G., Zhang H. (2007). Cellular microRNAs contribute to HIV-1 latency in resting primary CD4+ T lymphocytes. Nat. Med..

[55] Wen B.-P., Dai H.-J., Yang Y.-H., Zhuang Y., Sheng R. (2013). MicroRNA-23b inhibits enterovirus 71 replication through downregulation of EV71 VPl protein. Intervirology.

[56] Bai X.T., Nicot C. (2015). miR-28-3p is a cellular restriction factor that inhibits human T cell leukemia virus, type 1 (HTLV-1) replication and virus infection. J. Biol. Chem..

[57] Zhang G.-L., Li Y.-X., Zheng S.-Q., Liu M., Li X., Tang H. (2010). Suppression of hepatitis B virus replication by microRNA-199a-3p and microRNA-210. Antivir. Res..

[58] Zhang Z., Wu Q., Zhang T. (2020). Pangolin homology associated with 2019-nCoV. bioRxiv.

[59] Nao N., Yamagishi J., Miyamoto H., Igarashi M., Manzoor R., Ohnuma A., Tsuda Y., Furuyama W., Shigeno A., Kajihara M. (2017). Genetic predisposition to acquire a polybasic cleavage site for highly pathogenic avian influenza virus hemagglutinin. MBio.

[60] Hillen H.S., Kokic G., Farnung L., Dienemann C., Tegunov D., Cramer P. (2020). Structure of replicating SARS-CoV-2 polymerase. Nature..

[61] Yin W., Mao C., Luan X., Shen D.-D., Shen Q., Su H., Wang X., Zhou F., Zhao W., Gao M. (2020). Structural basis for inhibition of the RNA-dependent RNA polymerase from SARS-CoV-2 by remdesivir. Science.

[62] Sun J.-Z., Wang J., Wang S., Yuan D., Li Z., Yi B., Hou Q., Mao Y., Liu W. (2014). MicroRNA miR-320a and miR-140 inhibit mink enteritis virus infection by repression of its receptor, feline transferrin receptor. Virol. J..

[63] Duy J., Koehler J.W., Honko A.N., Schoepp R.J., Wauquier N., Gonzalez J.-P., Pitt M.L., Mucker E.M., Johnson J.C., O’Hearn A. (2016). Circulating microRNA profiles of Ebola virus infection. Sci. Rep..

[64] Domingo E., Holland J. (1997). RNA virus mutations and fitness for survival. Annu. Rev. Microbiol..

[65] Wang X.-S., Gong J.-N., Yu J., Wang F., Zhang X.-H., Yin X.-L., Tan Z.-Q., Luo Z.-M., Yang G.-H., Shen C. (2012). MicroRNA-29a and microRNA-142-3p are regulators of myeloid differentiation and acute myeloid leukemia. Blood.

[66] Lu X., Li X., He Q., Gao J., Gao Y., Liu B., Liu F. (2013). miR-142-3p regulates the formation and differentiation of hematopoietic stem cells in vertebrates. Cell Res..

[67] Wang F., Wang X.-S., Yang G.-H., Zhai P.-F., Xiao Z., Xia L.-Y., Chen L.-R., Wang Y., Wang X.-Z., Bi L.-X. (2012). miR-29a and miR-142-3p downregulation and diagnostic implication in human acute myeloid leukemia. Mol. Biol. Rep..

[68] Xu L., Xu Y., Jing Z., Wang X., Zha X., Zeng C., Chen S., Yang L., Luo G., Li B. (2014). Altered expression pattern of miR-29a, miR-29b and the target genes in myeloid leukemia. Exp. Hematol. Oncol..

[69] Park S.-Y., Lee J.H., Ha M., Nam J.-W., Kim V.N. (2009). miR-29 miRNAs activate p53 by targeting p85α and CDC42. Nat. Struct. Mol. Biol..

[70] Gong J., Yu J., Lin H., Zhang X., Yin X., Xiao Z., Wang F., Wang X., Su R., Shen C. (2014). The role, mechanism and potentially therapeutic application of microRNA-29 family in acute myeloid leukemia. Cell Death Differ..

[71] Xiong Y., Fang J.H., Yun J.P., Yang J., Zhang Y., Jia W.H., Zhuang S.M. (2010). Effects of MicroRNA-29 on apoptosis, tumorigenicity, and prognosis of hepatocellular carcinoma. Hepatology.

[72] Pekarsky Y., Croce C.M. (2015). Role of miR-15/16 in CLL. Cell Death Differ..

[73] Cimmino A., Calin G.A., Fabbri M., Iorio M.V., Ferracin M., Shimizu M., Wojcik S.E., Aqeilan R.I., Zupo S., Dono M. (2005). miR-15 and miR-16 induce apoptosis by targeting BCL2. Proc. Natl. Acad. Sci. USA.

[74] Ofir M., Hacohen D., Ginsberg D. (2011). MiR-15 and miR-16 are direct transcriptional targets of E2F1 that limit E2F-induced proliferation by targeting cyclin E. Mol. Cancer Res..

[75] Han H., Zhang Z., Yang X., Yang W., Xue C., Cao X. (2018). miR-23b suppresses lung carcinoma cell proliferation through CCNG1. Oncol. Lett..

[76] Luna J.M., Scheel T.K., Danino T., Shaw K.S., Mele A., Fak J.J., Nishiuchi E., Takacs C.N., Catanese M.T., de Jong Y.P. (2015). Hepatitis C virus RNA functionally sequesters miR-122. Cell.

[77] Fulzele S., Sahay B., Yusufu I., Lee T.J., Sharma A., Kolhe R., Isales C.M. (2020). COVID-19 Virulence in Aged Patients Might Be Impacted by the Host Cellular MicroRNAs Abundance/Profile. Aging Dis..

[78] Chow J.T.-S., Salmena L. (2020). Prediction and Analysis of SARS-CoV-2-Targeting MicroRNA in Human Lung Epithelium. Genes.

[79] Cullen B.R. (2006). Viruses and microRNAs. Nat. Genet..

[80] Demirci M.D.S., Adan A. (2020). Computational analysis of microRNA-mediated interactions in SARS-CoV-2 infection. PeerJ.

[81] Morales L., Oliveros J.C., Fernandez-Delgado R., Robert tenOever B., Enjuanes L., Sola I. (2017). SARS-CoV-encoded small RNAs contribute to infection-associated lung pathology. Cell Host Microbe.

[82] Arisan E.D., Dart A., Grant G.H., Arisan S., Cuhadaroglu S., Lange S., Uysal-Onganer P. (2020). The Prediction of miRNAs in SARS-CoV-2 Genomes: Hsa-miR Databases Identify 7 Key miRs Linked to Host Responses and Virus Pathogenicity-Related KEGG Pathways Significant for Comorbidities. Viruses.

[83] Periwal N., Sarma S., Arora P., Sood V. (2020). In-silico analysis of SARS-CoV-2 genomes: Insights from SARS encoded non-coding RNAs. bioRxiv.

[84] Choi E.-J., Kim H.B., Baek Y.H., Kim E.-H., Pascua P.N.Q., Park S.-J., Kwon H.-i., Lim G.-J., Kim S., Kim Y.-I. (2014). Differential microRNA expression following infection with a mouse-adapted, highly virulent avian H5N2 virus. BMC Microbiol..

[85] Zhang S., Ouyang X., Jiang X., Gu D., Lin Y., Kong S., Xie W. (2015). Dysregulated serum microRNA expression profile and potential biomarkers in hepatitis C virus-infected patients. Int. J. Med Sci..

[86] Ling T.-Y., Kuo M.-D., Li C.-L., Alice L.Y., Huang Y.-H., Wu T.-J., Lin Y.-C., Chen S.-H., Yu J. (2006). Identification of pulmonary Oct-4+ stem/progenitor cells and demonstration of their susceptibility to SARS coronavirus (SARS-CoV) infection in vitro. Proc. Natl. Acad. Sci. USA.

[87] Mallick B., Ghosh Z., Chakrabarti J. (2009). MicroRNome analysis unravels the molecular basis of SARS infection in bronchoalveolar stem cells. PLoS ONE.

[88] Kuba K., Imai Y., Rao S., Gao H., Guo F., Guan B., Huan Y., Yang P., Zhang Y., Deng W. (2005). A crucial role of angiotensin converting enzyme 2 (ACE2) in SARS coronavirus–induced lung injury. Nat. Med..

