# Supplementary material for: Computational Analysis of Targeting SARS-CoV-2, Viral Entry Proteins ACE2 and TMPRSS2, and Interferon Genes by Host MicroRNAs
Source: Genes (Basel). 2020 Nov 16;11(11):1354. doi: 10.3390/genes11111354 (PMC7696723; doi:10.3390/genes11111354)
Supplement: Supplementary file 1 [file genes-11-01354-s001.pdf]

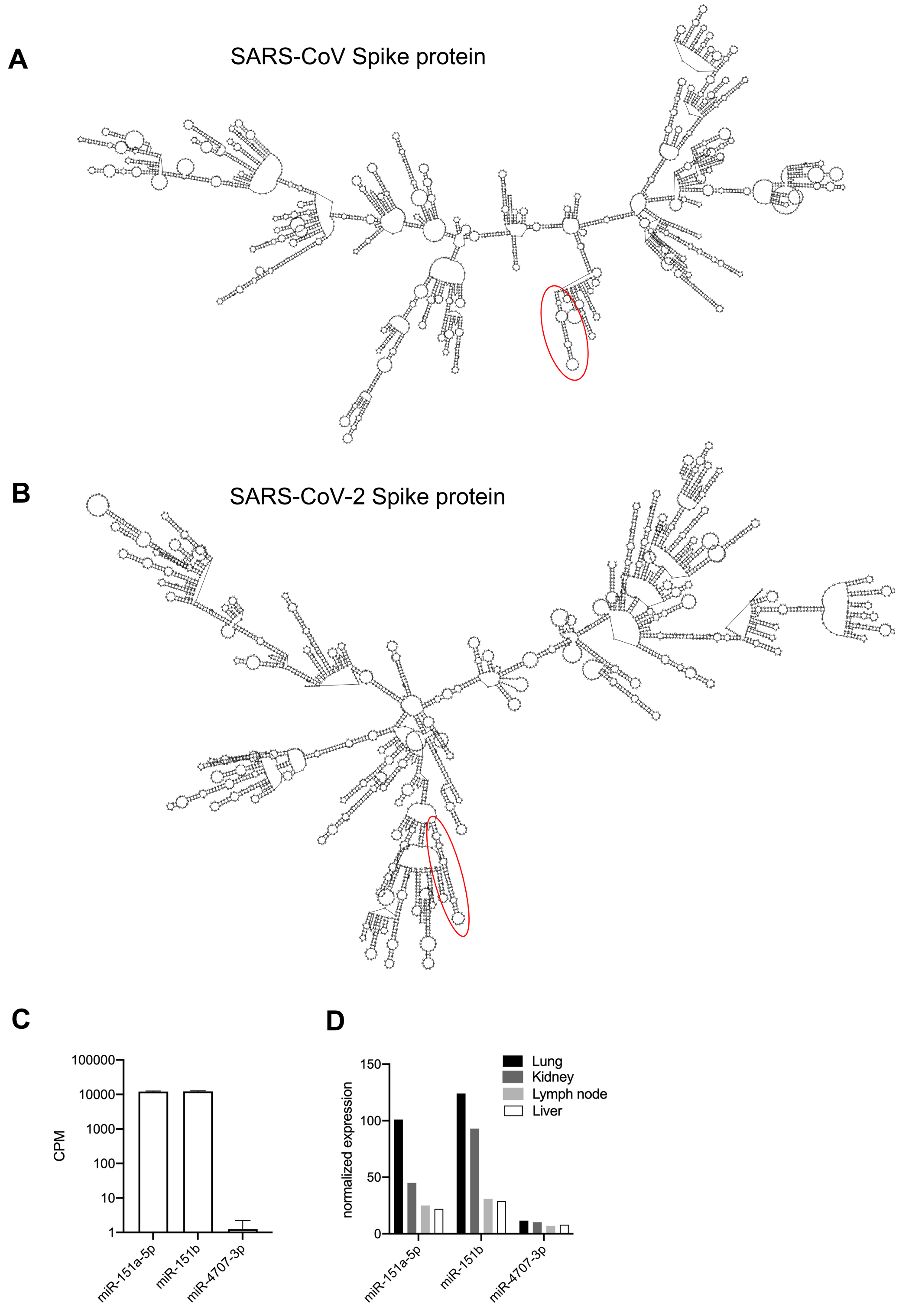

Supplemental Figure 1. Secondary structure of SARS-CoV and SARS-CoV-2 spike proteins and lung expression of miRNA binding in the PRRA region of the SARS-CoV-2 spike protein. Secondary structure of SARS-CoV **(A)** and SARS-CoV-2 **(B)** spike proteins and highlight of the spike regions of interest depicted in Figure 1C. **(C)** miR-151a-5p, -151b and -4707-3p expression in primary human lung fibroblasts. **(D)** Tissue ATLAS normalized expression of miR-151a-5p, -151b and -4707-3p in human lungs, kidney, lymph nodes, and liver.

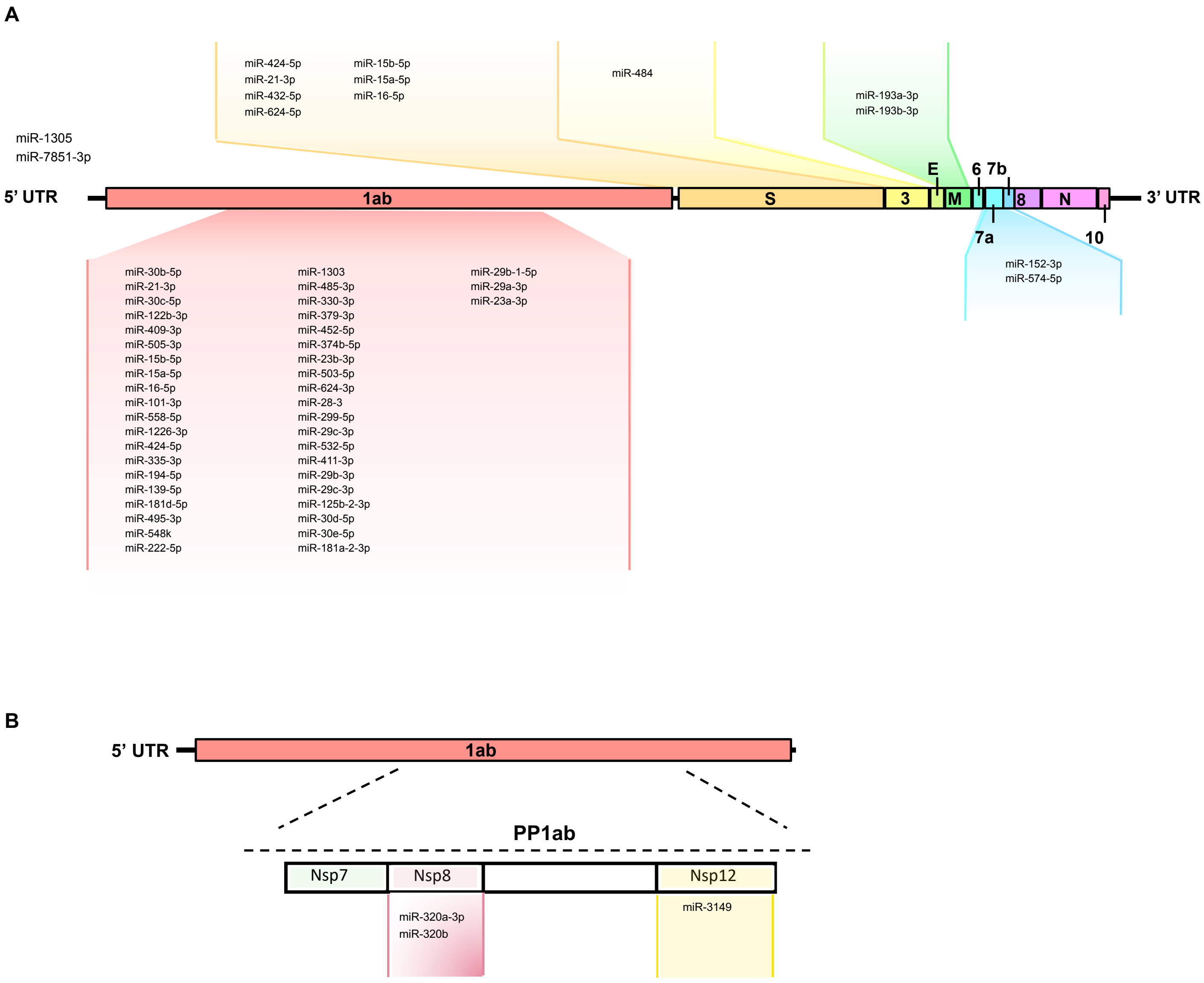

Supplemental Figure 2. Differential expression of miRNAs binding the SARS-CoV-2 genome between resistant versus susceptible cell lines. **(A)** MicroRNAs predicted to bind distinct regions of the SARS-CoV-2 genome that are differentially expressed between susceptible (Huh-7) and resistant (A549) cell lines ($p<0.2$); and **(B)** MicroRNAs predicted to bind in the Replicase polyprotein 1ab (PP1ab) region located within ORF1ab of SARS-CoV-2 that are differentially expressed between susceptible (Huh-7) and resistant (A549) cell lines ($p<0.2$).

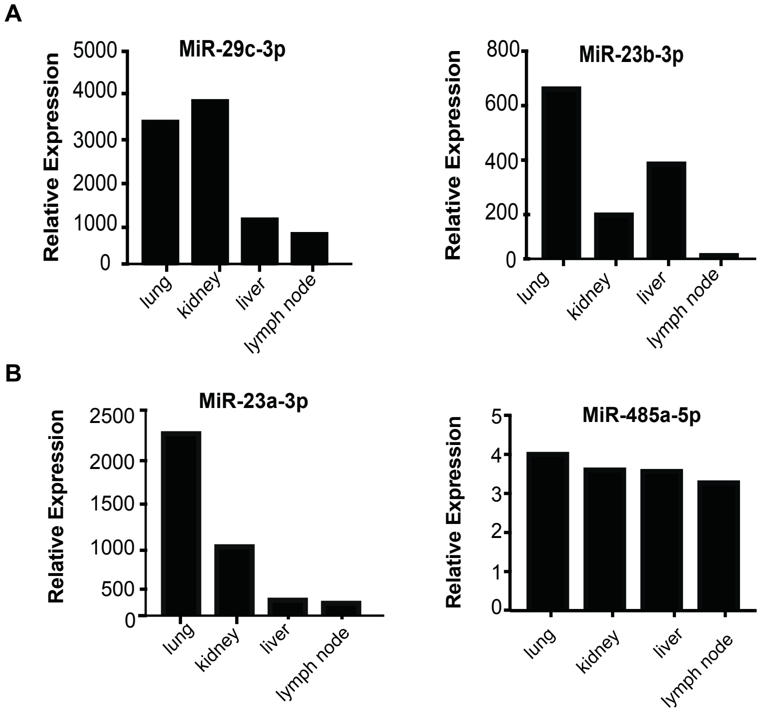

Supplemental Figure 3. Expression of the most differentially regulated miRNAs in relevant human tissues. Expression of top differentially regulated miRNAs between **(A)** A549 (resistant) and Huh7 (susceptible); and **(B)** human primary lung fibroblasts (LF, resistant) and Calu-3 (susceptible) cell lines in human lung, kidney, liver and lymph nodes.

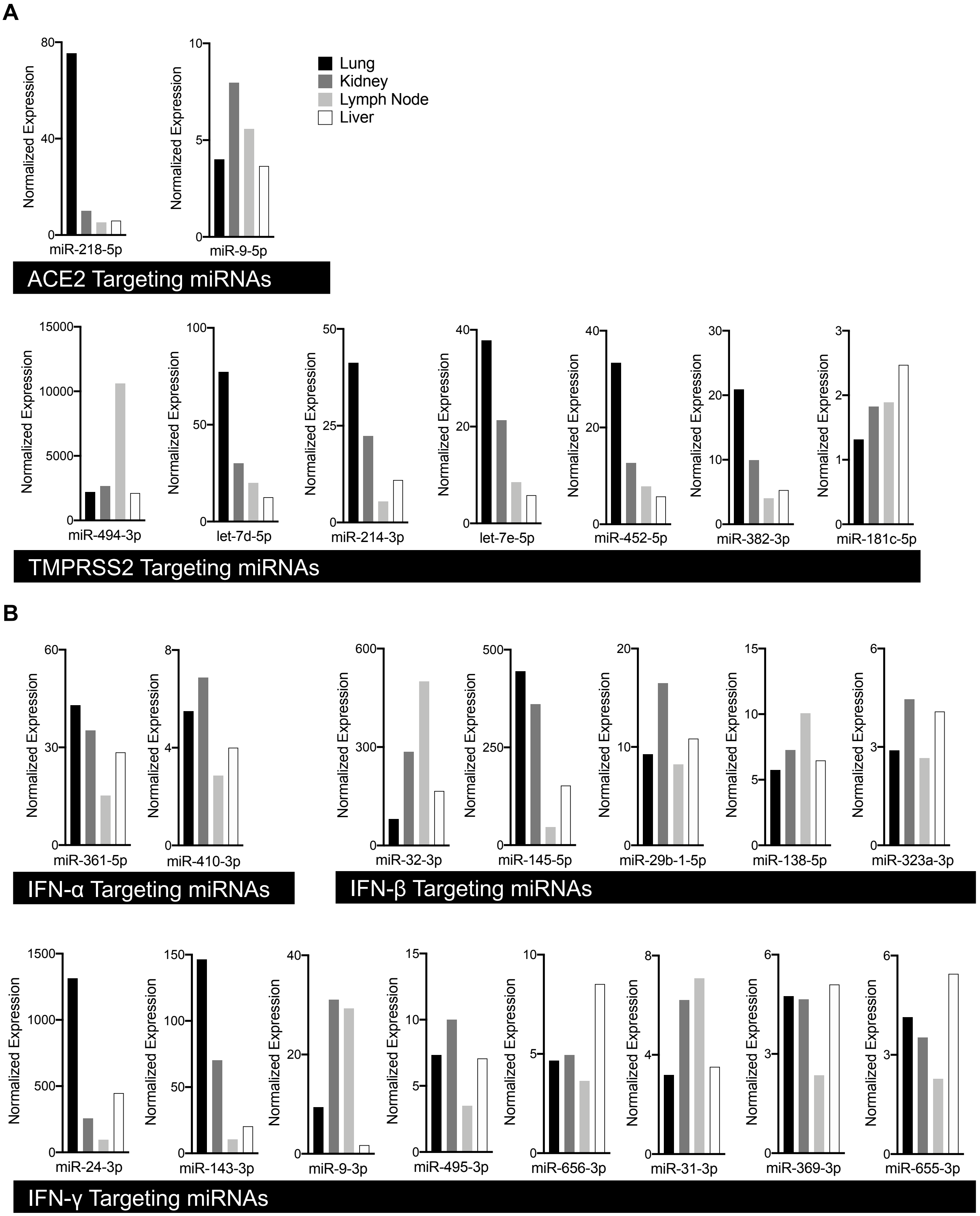

Supplemental Figure 4. Predicted miRNAs of host genes expression across tissues. **(A)** Expression of miRNAs predicted to target ACE2 and TMPRSS2 in human lungs, kidneys, lymph nodes, and liver. **(B)** Expression of miRNAs predicted to target IFN-α, IFN-β, and IFN-γ in human lungs, kidneys, lymph nodes, and liver. Normalized expression from Human miRNA tissue ATLAS.
